# Deafness‐Associated ADGRV1 Mutation Impairs USH2A Stability through Improper Phosphorylation of WHRN and WDSUB1 Recruitment

**DOI:** 10.1002/advs.202205993

**Published:** 2023-04-17

**Authors:** Ying Guan, Hai‐Bo Du, Zhao Yang, Yu‐Zhu Wang, Rui Ren, Wen‐Wen Liu, Chao Zhang, Jia‐Hai Zhang, Wen‐Tao An, Na‐Na Li, Xiao‐Xue Zeng, Jie Li, Yi‐Xiao Sun, Yan‐Fei Wang, Fan Yang, Jun Yang, Wei Xiong, Xiao Yu, Ren‐Jie Chai, Xiao‐Ming Tu, Jin‐Peng Sun, Zhi‐Gang Xu

**Affiliations:** ^1^ Key Laboratory Experimental Teratology of the Ministry of Education Department of Biochemistry and Molecular Biology School of Basic Medical Sciences Cheeloo College of Medicine Shandong University Jinan 250012 China; ^2^ Shandong Provincial Key Laboratory of Animal Cells and Developmental Biology Shandong University School of Life Sciences Qingdao 266237 China; ^3^ Air Force Medical Center PLA Beijing 100142 China; ^4^ MOE Key Laboratory for Membraneless Organelles and Cellular Dynamics Hefei National Research Center for Interdisciplinary Sciences at the Microscale School of Life Sciences University of Science and Technology of China Hefei 230022 China; ^5^ Department of Otolaryngology‐Head and Neck Surgery Shandong Provincial ENT Hospital Cheeloo College of Medicine Shandong University Jinan 250014 China; ^6^ Advanced Medical Research Institute Cheeloo College of Medicine Shandong University Jinan 250012 China; ^7^ School of Life Sciences IDG/McGovern Institute for Brain Research at Tsinghua Tsinghua University Beijing 100084 China; ^8^ Key Laboratory Experimental Teratology of the Ministry of Education Department of Physiology School of Basic Medical Sciences Cheeloo College of Medicine Shandong University Jinan 250012 China; ^9^ Department of Ophthalmology and Visual Sciences Moran Eye Center University of Utah Salt Lake City UT 84132 USA; ^10^ MOE Key Laboratory for Developmental Genes and Human Disease Institute of Life Sciences Jiangsu Province High‐Tech Key Laboratory for Bio‐Medical Research Southeast University Nanjing 210096 China; ^11^ Department of Physiology and Pathophysiology School of Basic Medical Sciences Peking University Key Laboratory of Molecular Cardiovascular Science Ministry of Education Beijing 100191 China; ^12^ Shandong Provincial Collaborative Innovation Center of Cell Biology Shandong Normal University Jinan 250014 China

**Keywords:** adhesion G protein‐coupled receptor V subfamily member 1, ankle link complex, deafness, phosphorylation, ubiquitination

## Abstract

The ankle‐link complex (ALC) consists of USH2A, WHRN, PDZD7, and ADGRV1 and plays an important role in hair cell development. At present, its architectural organization and signaling role remain unclear. By establishing *Adgrv1* Y6236fsX1 mutant mice as a model of the deafness‐associated human Y6244fsX1 mutation, the authors show here that the Y6236fsX1 mutation disrupts the interaction between adhesion G protein‐coupled receptor V subfamily member 1 (ADGRV1) and other ALC components, resulting in stereocilia disorganization and mechanoelectrical transduction (MET) deficits. Importantly, ADGRV1 inhibits WHRN phosphorylation through regional cAMP‐PKA signaling, which in turn regulates the ubiquitination and stability of USH2A via local signaling compartmentalization, whereas ADGRV1 Y6236fsX1 does not. Yeast two‐hybrid screening identified the E3 ligase WDSUB1 that binds to WHRN and regulates the ubiquitination of USH2A in a WHRN phosphorylation‐dependent manner. Further FlAsH‐BRET assay, NMR spectrometry, and mutagenesis analysis provided insights into the architectural organization of ALC and interaction motifs at single‐residue resolution. In conclusion, the present data suggest that ALC organization and accompanying local signal transduction play important roles in regulating the stability of the ALC.

## Graphical abstract

The ankle link complex (ALC) is essential for hair cell development and hearing. However, the architectural organization and signaling role of ALC remain unclear. Here, the authors show that ALC component ADGRV1 inhibits whirlin phosphorylation and regulates the stability of USH2A through compartmentalized cAMP‐PKA signaling. These results provide insights into the molecular mechanism underlying ALC assembly and dynamic regulation.

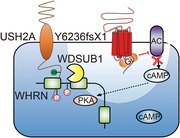

## 1 Introduction

The adhesion G protein‐coupled receptor V subfamily member 1 (ADGRV1), previously known as very large G protein‐coupled receptor 1 (Vlgr1), neuroepithelium‐notable (Neurepin), monogenic audiogenic seizure‐susceptible (Mass1) or G protein‐coupled receptor 98 (GPR98), is the largest G protein‐coupled receptor (GPCR) and plays important roles in the sensory and central nervous systems.^[^ ^1^ ^]^ Mutations in the human *ADGRV1* gene are associated with Usher syndrome type II, characterized by combined congenital deafness and blindness.^[^ ^2^ ^]^ In addition, a nonsense mutation in the *ADGRV1* gene was reported to be associated with febrile and afebrile seizures.^[^ ^3^ ^]^ Consistently, mutation or disruption of the gene *Adgrv1* causes deafness and audiogenic seizures in mice.^[^ ^4^ ^]^

ADGRV1 is expressed ubiquitously in various tissues, including the brain, lung, kidney, and eye, in addition to the inner ear.^[^ ^2^, ^4^, ^5^ ^]^ In the inner ear, ADGRV1 is localized at the base of hair cell stereocilia, forming the so‐called ankle link.^[^ ^4^, ^6^ ^]^ Stereocilia are actin‐based protrusions at the apical surface of hair cells that are indispensable for mechanoelectrical transduction (MET).^[^ ^7^ ^]^ Several types of extracellular links connect stereocilia and are important for maintaining stereociliary integrity.^[^ ^8^ ^]^ Among these extracellular links, ankle links are web‐like links that run parallel to the apical surface of hair cells, connecting the ankle regions of each neighboring stereocilium.^[^ ^8^ ^]^ In *Adgrv1* mutant or knockout mice, ankle links are disrupted, resulting in stereocilia disorganization and hearing loss.^[^ ^4^, ^6^, ^9^ ^]^ PDZD7, usherin (USH2A), and whirlin (WHRN) have been shown to localize at the ankle region of stereocilia and, together with ADGRV1, form the so‐called ankle‐link complex (ALC).^[^ ^6^, ^10^ ^]^

Although structurally considered as a GPCR, ADGRV1 had not been shown to mediate G protein signal transduction until recently.^[^ ^11^ ^]^ The selective combination of various extracellular domains with transmembrane regions and the C‐terminal tail of ADGRV1 was shown to activate G*α*s and G*α*q, resulting in increased intracellular cAMP levels and PKC phosphorylation.^[^ ^11b^ ^]^ In our previous work, we showed that ADGRV1 undergoes autocleavage, and the resultant transmembrane fragment constitutively activates G*α*i and blocks forskolin (FSK)‐induced cAMP elevation, which could be inhibited by PDZD7.^[^ ^11a^ ^]^ A deafness‐associated human *ADGRV1* mutation, Y6244fsX1, causes premature translation termination and produces a truncated protein without the C‐terminal 63 amino acids (aa).^[^ ^2^ ^]^ The corresponding mouse mutant, ADGRV1 Y6236fsX1, exhibits enhanced constitutive G*α*i activity that is no longer inhibited by PDZD7, which might be due to the absence of an interaction between the mutant ADGRV1 and PDZD7.^[^ ^11a^ ^]^

Despite the important structural role of ADGRV1 as a key ALC component, the physiological role of ADGRV1‐mediated signaling has not been fully elucidated regarding ALC formation and the dynamic regulation of the ALC. Furthermore, the detailed architecture of ALC is elusive, and the scaffolding role of this complex in ALC signaling remains a mystery. The deafness‐related A*dgrv1* Y6236fsX1 mutant, which remains mostly structurally intact but causes disruption of the ALC and alters downstream Gi signaling, serves as a valuable model to probe the dynamic regulation of the ALC and the role of ADGRV1‐mediated signaling. Therefore, to further explore the physiological role of ADGRV1‐mediated signal transduction and ALC formation in the inner ear, A*dgrv1* Y6236fsX1 mutant mice were established in the present work. The Y6236fsX1 mutation causes profound hearing loss in mice, recapitulating its deafness phenotype in humans. Our combined physiological, biochemical, and structural experiments not only revealed that ADGRV1‐mediated local signal transduction plays important roles in regulating the stability of ALC but also revealed that phosphorylation by PKA and ubiquitination mediated by WD repeat, SAM, and U‐box domain‐containing protein 1 (WDSUB1) serve as important mechanisms underlying dynamic ALC regulation.

## 2 Results

### 2.1 *Adgrv1* Y6236fsX1 Mutant Mice are Profoundly Deaf

**Figure 1:**
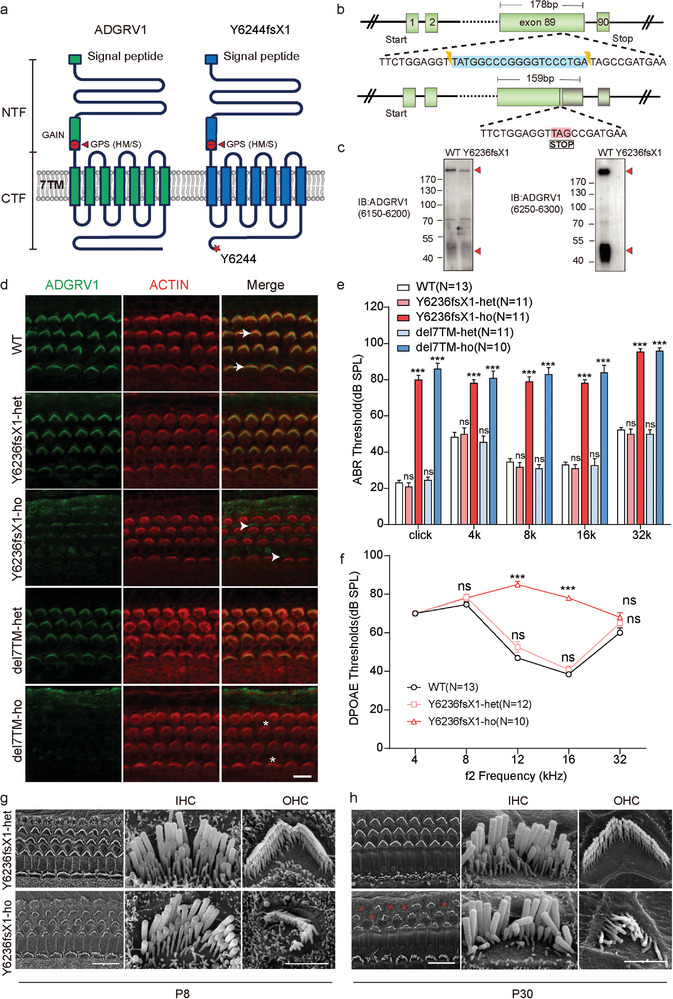
*Adgrv1* Y6236fsX1 mutation leads to hearing loss and stereocilia disorganization in mice. a) Schematic diagrams showing the sequence features of wide‐type (WT) human ADGRV1 and Y6244fsX1 mutant. The ADGRV1 Y6244fsX1 mutation leads to a premature translational termination, which results in the truncation of the C‐terminal 63 residues. b) Schematic representation of the generation of *Adgrv1* Y6236fsX1 mice using the CRISPR/Cas9 strategy. A deletion of 19 bp was introduced into exon 89 of the mouse *Adgrv1* gene, resulting in a frameshift and premature translational termination. c) Representative blotting showing endogenous ADGRV1 in the cochlea of WT or *Adgrv1* Y6236fsX1 mice. A band with > 200 kDa molecular weight corresponding to the intact ADGRV1 (or an endogenous isoform) and a 45‐kDa band corresponding to the ADGRV1‐CTF subunit were detected by both antibodies in the WT mice. In the Y6236fsX1 mutant, bands corresponding to truncated ADGRV1 were detected by the antibody recognizing the sequence (residue 6150–6200) before the truncating site but not the other antibody recognizing the C‐terminus (residue 6250–6300). Representative blotting from three independent experiments were shown. d) Cochlear whole mounts from P1 mice of indicated genotypes were stained with an anti‐ADGRV1 antibody specifically recognizing its N‐terminal extracellular fragment. TRITC‐phalloidin was used to visualize the F‐actin core of stereocilia. ADGRV1 immunoreactivity in the stereocilia of WT mice is indicated by arrows; ADGRV1 immunoreactivity in the apical bare zone of hair cells in the mutant mice is indicated by arrowheads; absence of ADGRV1 immunoreactivity at the corresponding position in the knockout mice is indicated by asterisks. All images were taken from the basal turn of the basilar membrane using a confocal microscope. Scale bar: 10 µm. Representative images from three independent experiments was shown. e) ABR thresholds of 2‐week‐old mice for click or pure tone stimuli. The number of animals used in each group was indicated in the brackets. f) DPOAE thresholds for each representative f2 frequency of P60 mice of different genotypes as indicated. The number of animals used in each group was indicated in the brackets. g,h) Representative SEM images showing hair bundle morphology of *Y6236fsX1* heterozygous or homozygous mice at g) P8 or h) P30. Images were taken from the middle turn of the auditory sensory epithelia. Asterisks indicate complete loss of hair bundles. IHC, inner hair cell; OHC, outer hair cell. Scale bar: 10 µm (in low magnification images) or 2 µm (in high magnification images). Representative images from three independent experiments were shown. Data information: e–f) ***p < 0.001; ns, no significant difference; *Adgrv1*/del7TM or Y6236fsX1 mutant mice compared with WT mice. The bars indicate the mean ± SEM values. Data were statistically analyzed using one‐way ANOVA with Dunnett's post hoc test.

The *ADGRV1* gene is highly conserved in the human and mouse genomes. The human *ADGRV1* Y6244fsX1 mutation is caused by a 19‐base pair (bp) deletion that results in premature translational termination (**Figure** **1**a). This frameshift mutation is associated with sensorineural hearing loss and retinitis pigmentosa^[^ ^1^, ^2^ ^]^ (Figure S1a, Supporting Information). To recapitulate the clinical phenotypes of the *ADGRV1* Y6244fsX1 human mutation in an animal model and explore the underlying mechanisms, we generated *Adgrv1* mutant mice using the CRISPR/Cas9 technique. Deletion of the corresponding 19 nucleotides in the mouse *Adgrv1* gene resulted in the Y6236fsX1 mutation, which produces a truncated ADGRV1 protein lacking the C‐terminal 63 aa, comparable to the effects of the human Y6244fsX1 mutation (Figure 1b and Figure S1b, Supporting Information). Genotyping PCR, Sanger sequencing, and Western blotting confirmed the successful construction of the mutant mice (Figure 1c and Figure S1c–e, Supporting Information).

Both heterozygous and homozygous *Adgrv1* Y6236fsX1 mice were viable and exhibited a normal life expectancy. The mutant mice had a normal body weight and showed no significant motor impairment, as revealed by gait analysis (Figure S1f–h, Supporting Information). The protein expression of ADGRV1 in the cochlea was further examined by whole‐mount immunostaining, which showed that ADGRV1 immunoreactivity was localized in the stereocilia of wild‐type (WT) or heterozygous *Adgrv1* Y6236fsX1 mice at postnatal day 1 (P1) (Figure 1d). However, ADGRV1 immunoreactivity was not detected in the stereocilia of homozygous *Adgrv1* Y6236fsX1 mice; instead, it localized at the apical surface of the hair cells, especially at the basal turn, suggesting that the Y6236fsX1 mutation affects the trafficking of ADGRV1 to the stereocilia (Figure 1d). Mislocalization of ADGRV1 is not observed in homozygous *Adgrv1* knockout mice (*Adgrv1*/del7TM) used as a control, in which seven transmembrane and cytoplasmic domains of ADGRV1 are deleted^[^ ^4b^ ^]^ (Figure 1d and Figure S1i, Supporting Information).

The hearing threshold of *Adgrv1* Y6236fsX1 mutant mice was then evaluated by auditory brainstem response (ABR) measurements. By the age of 2 weeks, homozygous *Adgrv1* Y6236fsX1 mutant mice showed a ∼60 dB hearing threshold elevation evoked by click stimuli, comparable to that observed for homozygous *Adgrv1*/del7TM knockout mice, whereas heterozygous Y6236fsX1 mutant mice and WT mice showed a normal threshold (Figure 1e). When pure‐tone stimuli were used, both Y6236fsX1 mutant mice and knockout mice showed a profound threshold elevation at frequencies from 4 to 32 kHz (Figure 1e). Distortion product otoacoustic emission (DPOAE) measurements were then carried out to examine the function of outer hair cells (OHCs) in the homozygous Y6236fsX1 mutant mice, which revealed significant threshold elevation of nearly 40 dB at 12 and 16 kHz (Figure 1f), suggesting that OHC function was compromised in Y6236fsX1 mutant mice. Taken together, these data suggest that *Adgrv1* Y6236fsX1 mutant mice are profoundly deaf and thus serve as a useful animal model for studying *ADGRV1* Y6244fsX1 mutation‐associated deafness.

### 2.2 *Adgrv1* Y6236fsX1 Mutation Leads to Stereocilia Disorganization

We then examined the morphology of cochlear hair cell stereocilia in *Adgrv1* Y6236fsX1 mutant mice using confocal microscopy to image phalloidin‐stained cochlear whole mounts. At P4, OHC stereocilia of homozygous *Adgrv1* Y6236fsX1 mutant mice exhibited a less‐defined V‐shape compared to that of heterozygous and WT mice, lacking bilateral symmetry and showing disorientation (Figure S2a–c, Supporting Information). At P14, the stereocilia disorganization in homozygous mutant OHCs became more profound (Figure S2a′–c′, Supporting Information). Stereocilia disorganization was further exacerbated at P28, and complete loss of OHC stereocilia was occasionally observed at this age (Figure S2a″–c″, Supporting Information). As a control, homozygous *Adgrv1*/del7TM knockout mice exhibited stereocilia disorganization similar to that of homozygous *Adgrv1* Y6236fsX1 mice (Figure S2d–d″, S2e–e″, Supporting Information).

Scanning electron microscopy (SEM) was then employed to examine the morphology of hair cell stereocilia at a higher resolution. When examined at P8, OHC stereocilia of homozygous *Adgrv1* Y6236fsX1 mutant mice were clearly disorganized and exhibited a poorly defined V‐shape and reduced bilateral symmetry, whereas the inner hair cell (IHC) stereocilia were largely normal (Figure 1g). By P30, the disorganization of OHC stereocilia in homozygous *Adgrv1* Y6236fsX1 mutant mice had become more profound, and some OHCs completely lost their hair bundles (Figure 1h). The mutant IHC stereocilia at this age still had a normal overall appearance, but degeneration of the third‐row mechanosensitive stereocilia was observed in some mutant IHCs (Figure 1h). Taken together, our data suggest that the stereocilia of cochlear hair cells became progressively disorganized in *Adgrv1* Y6236fsX1 mutant mice.

### 2.3 MET Currents are Decreased in *Adgrv1* Y6236fsX1 Cochlear Hair Cells

**Figure 2:**
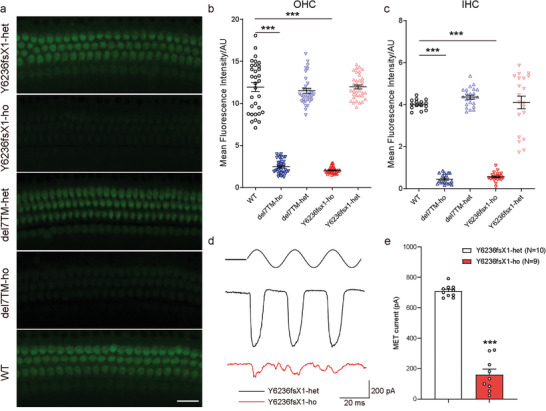
*Adgrv1* Y6236fsX1 mutation leads to impairment of MET functions. a) FM1‐43FX dye uptake by auditory hair cells from P5 mice of different genotypes as indicated. Images were taken from the middle turn of the cochlea using a confocal microscope. Scale bar: 20 µm. Representative images from three independent experiments were shown. b,c) Relative fluorescence intensity in b) OHCs and c) IHCs from mice of different genotypes were measured and analyzed using ImageJ software. Data are correlated with Figure 2a. Data were obtained from 15–23 OHCs or 32–38 IHCs from three mice in each group. d) Representative saturating MET currents in OHC from P6 *Y6236fsX1* heterozygous or homozygous mice triggered by a fluid jet system. e) Statistics of saturating MET currents in OHCs from P6 *Y6236fsX1* heterozygous or homozygous mice. Data are correlated to Figure 2d. Data information: b,c) ***p < 0.001; *Adgrv1*/del7TM or Y6236fsX1 mutant mice compared with WT mice. e) ***p < 0.001; homozygous *Adgrv1* Y6236fsX1 mice compared with heterozygous *Adgrv1* Y6236fsX1 mice. The bars indicate the mean ± SEM values. Data were statistically analyzed using b,c) one‐way ANOVA with Dunnett's post‐hoc test or e) Student's *t‐*test.

Stereocilia disorganization might affect the MET function of hair cells. When applied briefly, the fluorescent dye FM1‐43 and its fixable analog, FM1‐43FX, can enter hair cells through MET channels and thus serve as convenient indicators of the functional integrity of hair cells.^[^ ^12^ ^]^ We, therefore, performed an FM1‐43FX uptake experiment to examine the MET function of *Adgrv1* Y6236fsX1 mutant hair cells. The results showed that FM1‐43FX uptake at P5 was reduced by more than 80% in both OHCs and IHCs of homozygous *Adgrv1* Y6236fsX1 mice compared to WT and heterozygous control mice (**Figure** **2**a–c). Similar results were observed in homozygous *Adgrv1*/del7TM knockout mice (Figure 2a–c).

We next performed hair cell electrophysiology to evaluate the MET function of *Adgrv1* Y6236fsX1 mutant hair cells. The saturating MET currents of cochlear hair cells were recorded after the application of fluid jet stimulation to the hair bundles. A large MET current of 709.2 ± 12.5 pA was recorded in heterozygous *Adgrv1* Y6236fsX1 control OHCs at P6 (Figure 2d,e). In contrast, the maximum MET currents in homozygous *Adgrv1* Y6236fsX1 OHCs were reduced to 159.9 ± 36.5 pA (Figure 2d,e). Taken together, the results of the FM 1–43FX uptake experiment and hair cell electrophysiology recordings suggest that MET function is impaired in *Adgrv1* Y6236fsX1 cochlear hair cells.

### 2.4 *Adgrv1* Y6236fsX1 Mutation Disrupts ALC Formation

**Figure 3:**
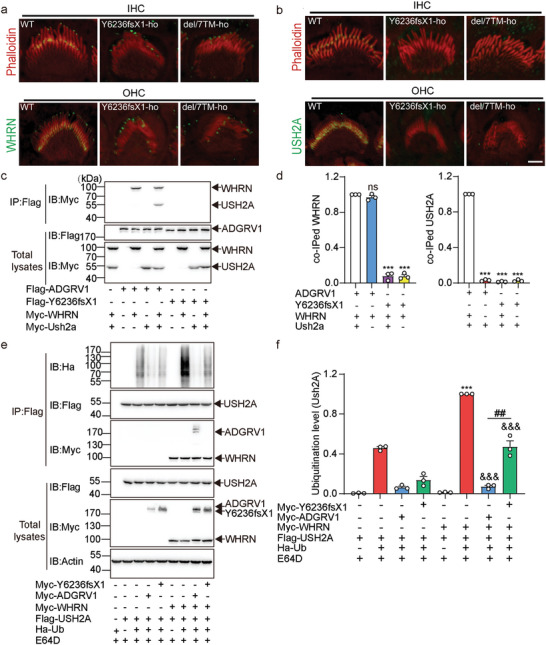
*Adgrv1* Y6236fsX1 mutation disrupts ALC formation. a,b) Whole‐mount immunostaining showing the localization of ALC components, a) WHRN and b) USH2A, in the stereocilia of IHC and OHC from P5 mice of different genotypes as indicated. TRITC‐phalloidin was used to visualize the F‐actin core of stereocilia. All images were taken from the middle turn of the basilar membrane using a confocal microscope. Scale bar: 2 µm. Representative images from three independent experiments were shown. c) Representative blotting showing coimmunoprecipitation of Flag‐ADGRV1 or Flag‐Y6236fsX1 with Myc‐WHRN and Myc‐USH2A in HEK293 cells. HEK293 cells were cotransfected with different combinations of ALC components as indicated in the figure. WT ADGRV1 or Y6236fsX1 mutant was immunoprecipitated by Flag antibody‐conjugated agarose, and the ALC components were detected by specific antibodies against Flag or Myc. d) Quantitative analysis of WHRN and USH2A levels that were coimmunoprecipitated with Flag‐ADGRV1. The data are correlated with Figure 3c and the band intensity of WHRN or USH2A co‐immunoprecipitated with Flag antibody‐conjugated agarose in the HEK293 cells transfected with Flag‐ADGRV1/Myc‐WHRN/Myc‐USH2A was used as the reference (normalized to 1). Data are from three independent experiments (*n* = 3). e) Representative blotting showing the ubiquitination levels of USH2A in HEK293 cells that were transfected with Myc‐WHRN, Flag‐USH2A, Myc‐ADGRV1 (WT or Y6236fsX1 mutant), and HA‐ubiquitin (HA‐UB) and were pretreated with lysosome inhibitor E64D. f) Quantitative analysis of the ubiquitination levels of Flag‐USH2A. The data are correlated with Figure 3e and the staining intensity of ubiquitinated USH2A (stain above 55 kd in the lane) in HEK293 cells transfected with Myc‐WHRN/Flag‐USH2A/HA‐Ub and treated with E64D was used as the reference (normalized to 1). Data are from three independent experiments (*n* = 3). Data information: d) ***p < 0.001; ns, no significant difference; HEK293 cells cotransfected with the other combinations of ALC components compared with those cotransfected with WT ADGRV1, USH2A, and WHRN. f) ***p < 0.001; HEK293 cells cotransfected with WHRN/USH2A/HA‐UB compared with those transfected with USH2A/HA‐UB. ^&&&^ *p* < 0.001; HEK293 cells transfected with ADGRV1/WHRN/USH2A/HA‐UB compared with those transfected with WHRN/USH2A/HA‐UB. ^##^ *p* < 0.01; HEK293 cells transfected with Y6236fsX1 compared with those transfected with WT ADGRV1. The bars indicate the mean ± SEM values. All data were statistically analyzed using one‐way ANOVA with Dunnett's post‐hoc test.

The loss of ADGRV1 immunoreactivity in the stereocilia of homozygous *Adgrv1* Y6236fsX1 cochlear hair cells suggests that ankle links are disrupted in these cells, which might contribute to stereocilia disorganization. We then performed whole‐mount immunostaining to examine the localization of other known ALC components in *Adgrv1* Y6236fsX1 hair cells. As reported previously,^[^ ^6^, ^10^ ^]^ PDZD7, WHRN, and USH2A immunoreactivity were detected at the ankle region of stereocilia in WT IHCs and OHCs, with additional WHRN immunoreactivity observed at the stereociliary tips (**Figure** **3**a,b and Figure S3a, Supporting Information). In homozygous *Adgrv1* Y6236fsX1 IHCs and OHCs, however, PDZD7 immunoreactivity was reduced in the stereocilia, whereas USH2A immunoreactivity was almost completely absent in the stereocilia (Figure 3b and Figure S3a, Supporting Information). Similar to USH2A immunoreactivity, WHRN immunoreactivity was absent at the ankle region of stereocilia in homozygous *Adgrv1* Y6236fsX1 IHCs and OHCs, but its localization at the stereociliary tip persisted (Figure 3a). Similar localization changes in ALC components were observed in *Adgrv1*/del7TM knockout mice (Figure 3a,b, and Figure S3a, Supporting Information).

We then used an in vitro reconstitution system in HEK293 cells to investigate the mechanisms underlying the regulation of ALC formation. Several ADGRV1 isoforms have been identified, and the longest isoform (ADGRV1b) consists of more than 6000 aa, which handicaps the in vitro expression and reconstitution of this isoform.^[^ ^1b^ ^]^ Therefore, in the present work, we used a shorter native isoform, ADGRV1a, which corresponds to the C‐terminal 1966 aa of ADGRV1b and includes a large extracellular part, a seven‐transmembrane domain and the cytoplasmic tail.^[^ ^5^ ^]^ We performed coimmunoprecipitation (co‐IP) experiments to examine whether the ADGRV1 Y6236fsX1 mutant affects ALC formation. The results showed that Myc‐tagged WHRN was immunoprecipitated together with Flag‐tagged ADGRV1 but not the ADGRV1 Y6236fsX1 mutant, suggesting that deletion of the last 63 aa in the ADGRV1 Y6236fsX1 mutant disrupts the interaction between WHRN and ADGRV1 (Figure 3c,d). Moreover, Myc‐tagged USH2A was not immunoprecipitated together with Flag‐tagged ADGRV1 unless WHRN was present, which was consistent with previous reports that WHRN serves as the scaffold for the ternary complex through its concurrent binding to both ADGRV1 and USH2A^[^ ^13^ ^]^ (Figure 3c,d). Taken together, the present data suggest that ALC formation is affected by the *Adgrv1* Y6236fsX1 mutation.

The reduced WHRN and USH2A immunoreactivity in the stereocilia of homozygous *Adgrv1* Y6236fsX1 mice suggests that the *Adgrv1* Y6236fsX1 mutation might affect the stability of WHRN and/or USH2A. The degradation rate of WHRN and USH2A was then examined in cultured cells treated with the protein synthesis inhibitor cycloheximide (CHX). The results showed that WHRN was quite stable and that the stability of WHRN was not affected by the presence of WT ADGRV1 or the Y6236fsX1 mutant ( Figure S3b,c, Supporting Information). In contrast, USH2A underwent time‐dependent degradation in the presence of WHRN. Interestingly, whereas WT ADGRV1 increased the stability of USH2A, the Y6236fsX1 mutant mostly lost this ability ( Figure S3b,d, Supporting Information). Proteins are degraded through proteasomal or lysosomal pathways. To explore the mechanism underlying USH2A degradation, we examined the effects of the proteasome inhibitor MG132 and lysosome inhibitor E64D on USH2A stability. Notably, constitutive USH2A degradation was completely abolished by pretreatment with E64D but not MG132, suggesting lysosome pathway‐dependent degradation of USH2A (Figure S3e,f, Supporting Information). Because ubiquitination is a key posttranslational modification that regulates protein stability, we examined the ubiquitination level of USH2A when coexpressed with other ALC components. We found that USH2A was constitutively ubiquitinated, which was further aggravated by coexpression with WHRN (Figure 3e,f). Consistent with the results of the protein stability assay, overexpression of WT ADGRV1 reduced the ubiquitination level of USH2A by ∼90% (Figure 3e,f). Although overexpression of the Y6236fsX1 mutant also inhibited the ubiquitination of USH2A, the inhibitory effect was decreased by ∼60% compared to that of WT ADGRV1 when ADGRV1 and its Y6236fsX1 mutant were normalized to similar expression levels (Figure 3e,f, and Figure S3g, Supporting Information). Taken together, our data suggest that ADGRV1 stabilizes USH2A by forming the ADGRV1/WHRN/USH2A complex and inhibits the constitutive degradation of USH2A via a ubiquitin‐lysosomal pathway, but this ability is largely abolished in ADGRV1 Y6236fsX1.

### 2.5 ADGRV1 but not the Y6236fsX1 Mutant Inhibits PKA‐Mediated WHRN Phosphorylation and Increases USH2A Stability

**Figure 4:**
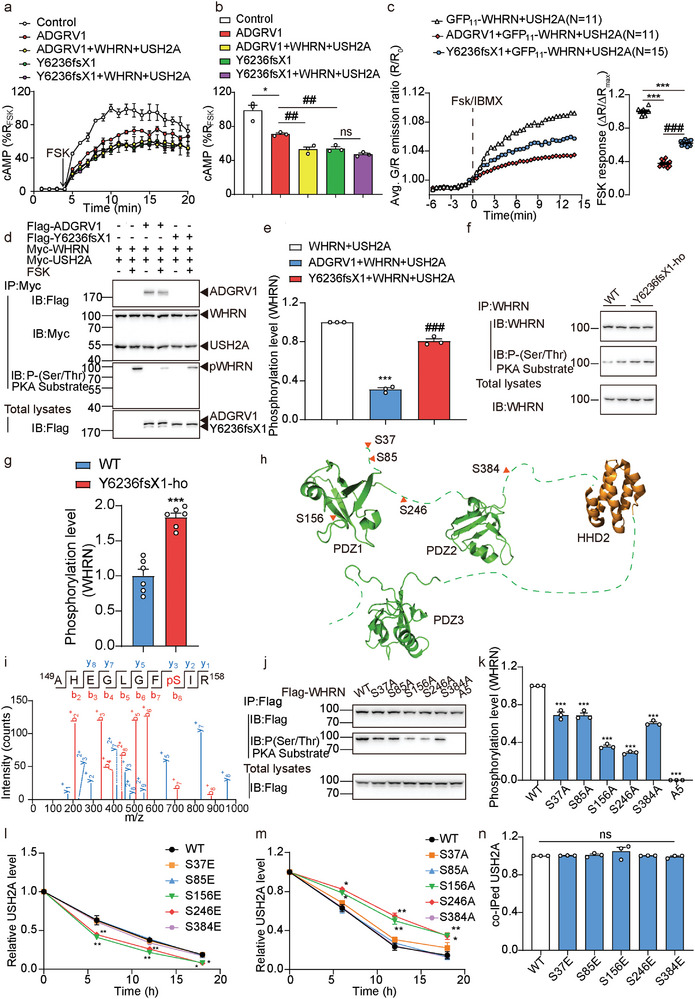
*Adgrv1* Y6236fsX1 mutation attenuates the inhibitory effects on local cAMP/PKA‐regulated WHRN phosphorylation. a,b) Effects of WT ADGRV1 or Y6236fsX1 mutant on the forskolin (FSK)‐induced cAMP accumulation in HEK293 cells in the absence or presence of WHRN and USH2A. Both the time course of a) cAMP production and b) quantitative analysis were shown. Data were normalized to the maximal cAMP response in control cells transfected with empty vector pcDNA3.1 and are from three independent experiments (*n* = 3). c) Left: Time course of cAMP production indicated by average G/R emission in HEK293 cells expressing WHRN‐FluoSTEP‐ICUE probe, USH2A and ADGRV1 (WT or Y6326fsX1 mutant) following stimulation with 50 µM Fsk and 100 µM IBMX. Right: Summary of emission ratio change (*△R/△R_max_*) for WHRN‐FluoSTEP‐ICUE (*△R/△R*_max_; see Experimental Section). Data were obtained from 11–15 cells from three independent experiments. The domain structure of the WHRN‐FluoSTEP‐ICUE probe was shown in Figure S4g, Supporting Information. d) Representative blotting showing the FSK‐stimulated phosphorylation levels of USH2A or WHRN in HEK293 cells transfected with Myc‐WHRN, Myc‐USH2A, and Flag‐ADGRV1 (WT or Y6236fsX1 mutant). HEK293 cells were cotransfected with different combinations of ALC components as indicated in the Figure. The cells were treated with 10 µM FSK or a control vehicle for 30 min before the lysates were immunoprecipitated by Myc antibody‐conjugated agarose. The phosphorylation levels of Myc‐tagged WHRN or USH2A were detected by phospho‐(Ser/Thr) PKA substrate antibody. Only the phosphorylation of WHRN but not USH2A was detected as indicated by the pWHRN bands. e) Quantitative analysis of FSK‐stimulated phosphorylation of WHRN in HEK293 cells. The data are correlated with Figure 4d and the band intensity of phosphorylated WHRN co‐immunoprecipitated with Myc antibody‐conjugated agarose in the HEK293 cells transfected with Myc‐WHRN/Myc‐USH2A and treated with 10 µM forskolin was used as the reference (normalized to 1). Data are from three independent experiments (*n* = 3). f) Representative blotting and g) quantitative analysis of WHRN phosphorylation levels in the lysates of cochlea isolated from WT or Y6236fsX1 mutant mice. The lysates were immunoprecipitated by WHRN antibody‐conjugated agarose and the phosphorylation levels were detected by phospho‐(Ser/Thr) PKA substrate antibody. Data were normalized to the WHRN phosphorylation level in the lysates of cochlea isolated from WT mice and are from three independent experiments (*n* = 3). h) The PKA phosphorylation sites on WHRN were identified by mass spectrometry analysis. HEK293 cells transfected with Flag‐WHRN were stimulated with 10 µM FSK or control vehicle for 30 min before the WHRN was immunoprecipitated by Flag antibody‐conjugated agarose. The bound proteins were eluted with buffer containing Flag peptide and subjected to mass spectrometry analysis. Data are correlated with Figure S5, Supporting Information. i) Mass spectrum of the WHRN peptide containing phosphorylated serine at S156. j) Representative blotting and k) quantitative analysis of PKA phosphorylation levels of WHRN in HEK293 cells transfected with Flag‐tagged WT WHRN or mutants. Data are normalized to the phosphorylation level of WT WHRN and are from three independent experiments (*n* = 3). l) Effects of different phospho‐mimic mutations or m) phospho‐deficient mutations of WHRN on the stability of USH2A. Data are correlated with Figure S4k,l, Supporting Information and are from three independent experiments (n = 3). n) Quantitative analysis of the Myc‐USH2A coimmunoprecipitated with Flag‐tagged WHRN (WT or phospho‐mimic mutants) in HEK293 cells. Data are correlated with Figure S4m, Supporting Information and are from three independent experiments (*n* = 3). Data information: b) *p < 0.05; HEK293 cells transfected with WT ADGRV1 compared with the control cells. ^##^ *p* < 0.01; HEK293 cells transfected with ADGRV1/WHRN/USH2A or with Y6236fsX1 compared with those transfected with WT ADGRV1. ns: no significant difference; HEK293 cells transfected with Y6236fsX1/WHRN/USH2A compared with those transfected with Y6236fsX1 only. c) ***p < 0.001; HEK293 cells transfected with WHRN‐FluoSTEP‐ICUE probe, USH2A, and ADGRV1 (WT or mutant) compared with those transfected with WHRN‐FluoSTEP‐ICUE probe and USH2A. ^###^ *p* < 0.001; HEK293 cells transfected with Y6236fsX1 compared with those transfected with WT ADGRV1. e) ***p < 0.001; HEK293 cells transfected with ADGRV1/WHRN/USH2A compared with those transfected with WHRN/USH2A. ^###^ *p* < 0.001; HEK293 cells transfected with Y6236fsX1/WHRN/USH2A compared with those transfected with ADGRV1/WHRN/USH2A. g) ***p < 0.001; Y6236fsX1 mutant mice compared with WT mice. k–n) *p < 0.05; **p < 0.01; ***p < 0.001; ns: no significant difference; HEK293 cells transfected with WHRN mutants compared with those transfected with WT WHRN. The bars indicate the mean ± SEM values. Data were statistically analyzed using b,c,e,g,k,n) one‐way or l,m) two‐way ANOVA with Dunnett's post‐hoc test.

To further decipher the underlying mechanisms by which ADGRV1 regulates USH2A stability, we examined G protein‐mediated signaling downstream of ADGRV1. Similar to other adhesion GPCR (aGPCR) family members, ADGRV1 undergoes constitutive autocleavage to produce two active fragments, an N‐terminal fragment (NTF) and a C‐terminal fragment (CTF, also referred to as the *β* subunit). Previously, we showed that ADGRV1‐CTF inhibits FSK‐induced cAMP elevation by coupling to G*α*i.^[^ ^11a^ ^]^ Here, we showed that full‐length ADGRV1a also inhibits FSK‐induced cAMP elevation, albeit with lower efficacy compared with that of ADGRV1‐CTF when expressed at similar levels in HEK293 cells ( Figure S4a *–*c, Supporting Information). Moreover, less WHRN was immunoprecipitated with ADGRV1a than with ADGRV1‐CTF when expressed at similar levels in HEK293 cells ( Figure S4a,b,d, Supporting Information). These results suggest an inhibitory effect of NTF on the constitutive signal transduction of ADGRV1, which has been shown in several aGPCRs.^[^ ^14^ ^]^ Importantly, coexpression of WHRN and USH2A enhanced the Gi activity of ADGRV1a by ∼25% (**Figure** **4**a,b). Although the ADGRV1a Y6236fsX1 mutant alone shows more robust Gi activity than WT ADGRV1a, this Y6236fsX1‐regulated cAMP inhibition could not be further enhanced by the coexpression of WHRN and USH2A, which was consistent with its inability to form a functional ternary complex with these two proteins (Figure 4a,b). Notably, the inhibitory effect of ADGRV1a WT or the Y6236fsX1 mutant on FSK‐stimulated cAMP production was completely abolished by pertussis toxin (PTX) preincubation, indicating specific Gi‐mediated signaling (Figure S4e,f, Supporting Information).

The comparable effects on FSK‐induced cAMP levels in transfected cells suggest that WT ADGRV1 and the Y6236fsX1 mutant have similar global Gi‐activating abilities. However, previous studies revealed that intracellular cAMP can be compartmentalized and that spatiotemporal cAMP signaling downstream of GPCRs is tightly controlled to ensure specific cellular responses.^[^ ^15^ ^]^ To examine potential ADGRV1‐mediated cAMP compartmentalization, we employed FluoSTEP, which is a genetically encoded fluorescent biosensor that combines self‐complementing split GFP and fluorescence resonance energy transfer (FRET) technology to report real‐time compartmentalized signaling dynamics.^[^ ^16^ ^]^ We used two sets of FluoSTEP probes, including actin‐FluoSTEP‐ICUE, which probes global cAMP signaling, and WHRN‐FluoSTEP‐ICUE, which probes compartmentalized cAMP signaling around WHRN (Figure S4g, Supporting Information). We found that when cotransfected with WHRN/USH2A and expressed at similar levels in HEK293 cells, WT ADGRV1 and Y6236fsX1 decreased the FSK‐induced cAMP to similar levels (Figure S4h, Supporting Information). However, the localized cAMP level around WHRN in cells transfected with the WT ADGRV1/WHRN/USH2A complex was significantly lower than that in cells transfected with the Y6236fsX1/WHRN/USH2A complex (Figure 4c). To further explore the functional outcomes of potential compartmentalized cAMP signaling, we examined the PKA‐mediated phosphorylation of WHRN and USH2A, which might reflect localized PKA activity downstream of cAMP and close to the ADGRV1/WHRN/USH2A complex. Notably, FSK‐induced, PKA‐mediated phosphorylation of WHRN, but not USH2A, was readily detected and was inhibited by ∼70% and 20% in HEK293 cells overexpressing WT ADGRV1 or the Y6236fsX1 mutant, respectively (Figure 4d,e). The FSK‐induced WHRN phosphorylation was PKA‐dependent since it was completely abrogated by pretreatment with the PKA inhibitor PKI (14‐22) but not by the PKC inhibitor Ro 31–8220 (Figure S4i,j, Supporting Information). Consistent with these in vitro data, the endogenous phosphorylation level of WHRN mediated by PKA in the cochlea of Y6236fsX1 mice was found to be ∼1.8‐fold higher than that of WT mice (Figure 4f,g). Using mass spectrometry analysis, we identified five potential PKA‐mediated phosphorylation sites in WHRN: S37, S85, S156, S246, and S384 (Figure 4h,i, and Figure S5, Supporting Information). Alanine substitution at any one of these five Ser residues impaired the PKA‐mediated phosphorylation level of WHRN, and combined alanine mutations completely abolished PKA‐mediated phosphorylation, indicating that these residues are PKA‐mediated phosphorylation sites (Figure 4j,k).

To further investigate the underlying mechanism by which WHRN phosphorylation regulates USH2A stability, we generated mutations to mimic phosphorylated WHRN and examined their effects on USH2A stability. The results showed that whereas the mutation of S156 or S246 to the phosphomimetic residue Glu significantly decreased the stability of USH2A, the replacement of these two resides with Ala led to the opposite effects, supporting a functional link between WHRN phosphorylation and USH2A stability (Figure 4l,m, Figure S4k,l, Supporting Information). However, the interaction between WHRN and USH2A was not affected by these mutations, as revealed by the co‐IP assay (Figure 4n and Figure S4m, Supporting Information). These results suggest that phosphorylated WHRN might regulate USH2A stability through an indirect mechanism.

Taken together, these results suggested that WT ADGRV1 may exert a stronger inhibitory effect than ADGRV1 Y6236fsX1 on cAMP‐PKA signaling localized around WHRN, which might contribute to the different effects on USH2A stability.

### 2.6 Phosphorylation of WHRN Regulates USH2A Ubiquitination by Recruiting the U‐box Ubiquitin Ligase WDSUB1

**Figure 5:**
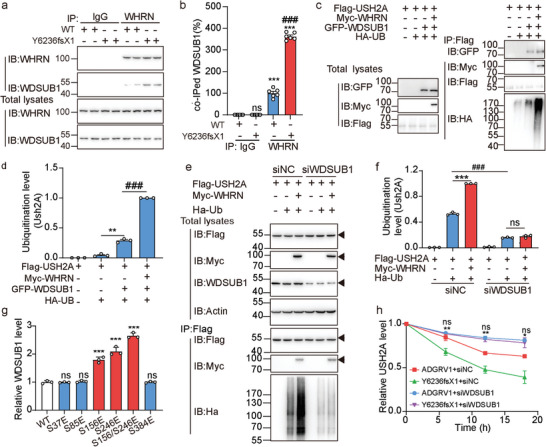
Phosphorylated WHRN regulates USH2A ubiquitination through recruiting WDSUB1. a) Representative blotting and b) quantitative analysis of the coimmunoprecipitation of WHRN with WDSUB1 in the lysates of cochlea isolated from WT or Y6236fsX1 mutant mice. Data are from three independent experiments (*n* = 3). c) Representative blotting and d) quantitative analysis of USH2A ubiquitination in HEK293 cells transfected with Flag‐USH2A and HA‐UB in the absence or presence of GFP‐WDSUB1 and Myc‐WHRN. Data are from three independent experiments (*n* = 3). e) Representative blotting and f) quantitative analysis of USH2A ubiquitination in HEK293 cells transfected with Flag‐USH2A, Myc‐WHRN, and HA‐UB in the presence of control or WDSUB1 siRNA. Data are from three independent experiments (*n* = 3). g) Quantitative analysis of the GFP‐WSDUB1 coimmunoprecipitated with Flag‐WHRN (WT or phospho‐mimic mutants) in HEK293 cells. Data are correlated with Figure S6h, Supporting Information and are from three independent experiments (*n* = 3). h) The stability of USH2A in ADGRV1 (WT or Y6236fsX1) overexpressed HEK293 cells that were treated with control or WDSUB1 siRNA. Data are correlated with Figure S6i, Supporting Information and are from three independent experiments (*n* = 3). Data information: b) ***p < 0.001; ns: no significant difference; WDSUB1 immunoprecipitated with WHRN compared with those immunoprecipitated with IgG. ^###^ *p* < 0.001; WDSUB1 immunoprecipitated in Y6236fsX1 mutant mice compared with that in WT mice. d) **p < 0.01; HEK293 cells transfected with USH2A/UB/WDSUB1 compared with those transfected with USH2A/UB. ^###^ *p* < 0.001; HEK293 cells transfected with USH2A/UB/WDSUB1/WHRN compared with those transfected with USH2A/UB/WDSUB1. f) ***p < 0.001; ns: no significant difference; HEK293 cells transfected with USH2A/WHRN/UB compared with those treated with the same siRNA but transfected only with USH2A/UB. ^###^ *p* < 0.001; HEK293 cells treated with siWDSUB1 compared with those treated with control siRNA. g) ***p < 0.001; ns, no significant difference; HEK293 cells transfected with WHRN mutants compared with those transfected with WT WHRN. h) *p < 0.05; **p < 0.01; HEK293 cells treated with siWDSUB1 compared with those treated with control siRNA. ns: no significant difference; HEK293 cells transfected with Y6236fsX1 mutant compared with those transfected with WT ADGRV1. The bars indicate the mean ± SEM values. All data were statistically analyzed using b,d,f,g) one‐way or h) two‐way ANOVA with Dunnett's post‐hoc test.

To identify the potential intermediary regulator between WHRN and USH2A, we performed yeast two‐hybrid screening of a cochlear cDNA library using WHRN as bait (Figure S6a, Supporting Information). A total of 18 positive clones were obtained, encoding seven candidate WHRN‐binding proteins (Figure S6b, Supporting Information). Among these candidates, the U‐box ubiquitin ligase WDSUB1, which is expressed in the stereocilia (near the base) and cell bodies of OHCs and IHCs, attracted our attention and was selected for further characterization (Figure S6c,d, Supporting Information). The interaction between WHRN and WDSUB1 was confirmed both in vitro and in vivo by co‐IP assay (**Figure** **5**a,b, and Figure S6e, Supporting Information). The specificity of the WDSUB1 antibody was verified by Western blot analysis in the heterologous expression system using other U‐box ubiquitin ligase family members, including CYC4 and UBOX5, as the negative control (Figure S6f, Supporting Information). Co‐expression and interaction of WDSUB1 with WHRN suggest that it might play a regulatory role in ALC dynamics. Consistent with this hypothesis, the overexpression of WDSUB1 in HEK293 cells significantly increased the constitutive ubiquitination of USH2A, which was further augmented by ≈2.5‐fold when WHRN was incorporated (Figure 5c,d). In contrast, the knockdown of WDSUB1 in HEK293 cells decreased the constitutive ubiquitination of USH2A by ≈70% and completely abolished the facilitating effect of WHRN (Figure 5e,f, and Figure S6g, Supporting Information). These results suggested that WDSUB1 acts as a regulator of USH2A ubiquitination through engagement with WHRN.

We next explored the potential regulatory effects of WHRN phosphorylation on the WHRN‐WDSUB1 interaction. We found that the interaction between WDSUB1 and WHRN was increased when S156 or S246 of WHRN was mutated to Glu, and both mutations were shown to reduce USH2A stability in vitro (Figures 4l, and 5g and Figure S6h, Supporting Information). Consistently, ≈2.5‐fold higher WDSUB1 was immunoprecipitated together with WHRN in the cochlear lysate of Y6236fsX1 mutant mice than in that of WT mice (Figure 5b). These findings implied that distinct WHRN phosphorylation statuses elicited by WT ADGRV1 and Y6236fsX1 might differentially recruit WDSUB1 and regulate USH2A stability. Consistent with this hypothesis, knockdown of WDSUB1 blunted the Y6236fsX1‐regulated degradation of USH2A in HEK239 cells, which showed comparable stability to USH2A coexpressed with WT ADGRV1 (Figure 5h and Figure S6i, Supporting Information).

The intracellular C‐terminus of USH2A contains seven lysine residues. To identify the lysine residue at which WHRN/WDSUB1‐mediated ubiquitination occurs, we generated a USH2A‐K0 mutant in which all seven intracellular lysine residues were mutated to arginine and then reintroduced each lysine residue or lysine cluster to examine the effects of these residues on WHRN/WDSUB1‐mediated USH2A ubiquitination. The results revealed that the back‐mutation R5075K or R5145K/R5146K significantly rescued USH2A ubiquitination (Figure S6j–l, Supporting Information). Collectively, these results suggested a model in which WHRN acts as a scaffold for both USH2A and WDSUB1 to regulate the ubiquitination levels of USH2A by WDSUB1.

### 2.7 Investigation of the Potential Interaction Mode of ALC by ITC and FlAsH‐BRET

**Figure 6:**
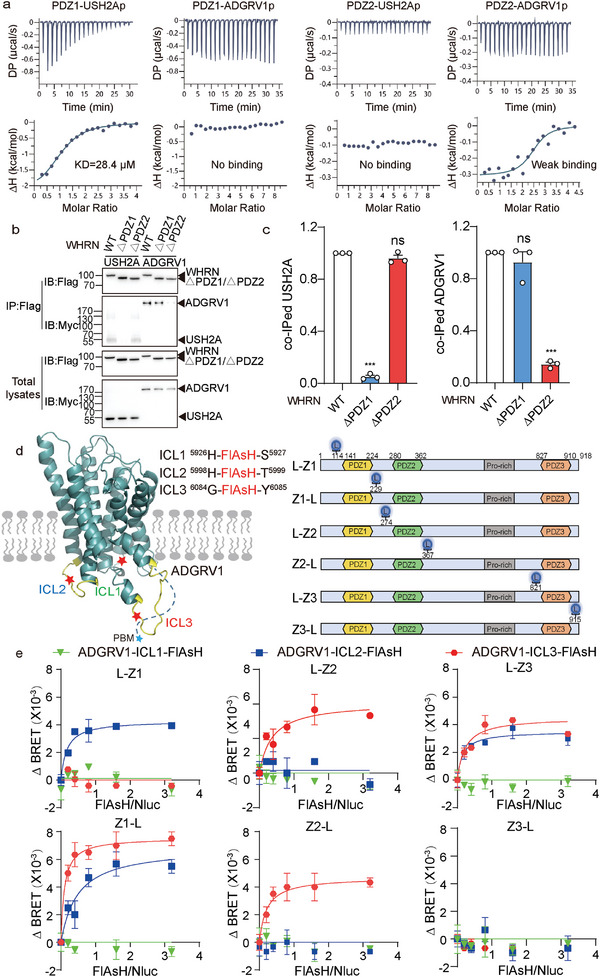
Potential interaction mode of the ALC revealed by ITC and FlAsH‐BRET. a) Interactions between the first two PDZ domains of WHRN and synthetic USH2A/ADGRV1 C‐terminal peptides revealed by isothermal titration calorimetry (ITC). The 10‐residue C‐terminal peptides of both ADGRV1 and USH2A harboring the PDZ‐binding motif (denoted as ADGRV1p and USH2Ap) were synthesized and titrated with purified WHRN‐PDZ1 and WHRN‐PDZ2 proteins. b) Representative blotting and c) quantitative analysis of the USH2A or ADGRV1 levels coimmunoprecipitated with the WT or PDZ domain‐truncated WHRN. Data are from three independent experiments (*n* = 3). d) Schematics illustrating the ADGRV1 with a FlAsH motif (CCPGCC) incorporated at corresponding sites in ICL1, ICL2, or ICL3 and the WHRN with a NanoLuc (Nluc) moiety inserted at distinct sites flanking the three PDZ domains (denoted as L‐Z1, Z1‐L, L‐Z2, Z2‐L, L‐Z3, and Z3‐L, respectively). e) Saturation BRET signal between NLuc and FlAsH in HEK293 cells co‐transfected with a fixed amount of NLuc‐WHRN plasmid and an increasing amount of ADGRV1‐FlAsH plasmids. Data are from three independent experiments (*n* = 3). Data information: c) ***p < 0.001; ns: no significant difference; HEK293 cells transfected with WHRN mutants compared with those transfected with WT WHRN. The bars indicate the mean ± SEM values. All data were statistically analyzed using one‐way ANOVA with Dunnett's post‐hoc test.

To further delineate the mode by which components of the ternary ADGRV1‐WHRN‐USH2A complex interact, we next investigated the architecture of ALC by isothermal titration calorimetry (ITC), surface plasmon resonance (SPR) and intermolecular FlAsH‐bioluminescence resonance energy transfer (FlAsH‐BRET), focusing on their known intermolecular interaction motifs and three intracellular loops (ICLs) in ADGRV1. Both ADGRV1 and USH2A contain a PDZ‐binding motif (PBM) at the intracellular C‐terminus, while WHRN contains 3 PDZ domains that are important for scaffolding PBM‐containing proteins. Although previous in vitro studies suggested that the first two PDZ domains of WHRN are required for the assembly of ADGRV1 and USH2A to form an intact ALC, how the PBMs of each receptor selectively interact with WHRN PDZ domains has not been characterized.^[^ ^13b^ ^]^ To explore the detailed mode by which these ALC components interact, we synthesized the 10‐residue C‐terminal tail peptides of ADGRV1 and USH2A harboring the PBM (denoted ADGRV1p and USH2Ap, respectively) and titrated the purified WHRN‐PDZ1 domain and WHRN‐PDZ2 domain with the synthetic peptides to measure their dissociation constant by ITC. The results showed that USH2Ap bound WHRN‐PDZ1 with an affinity of 28.4 ± 4.2 µM but exhibited no measurable binding to WHRN‐PDZ2 (**Figure** **6**a). In contrast, ADGRV1p showed weak binding to WHRN‐PDZ2 but failed to bind to WHRN‐PDZ1 (Figure 6a). These results are further supported by SPR data that revealed the measured dissociation constant (*Kd*) for USH2Ap and WHRN‐PDZ1 to be 23.00 ± 2.01 µM and that for ADGRV1p and WHRN‐PDZ2 to be 61.20 ± 4.52 µM (Figure S7a, Supporting Information). Consistently, the deletion of PDZ1 from WHRN eliminated the interaction between USH2A and WHRN in HEK293 cells but had no significant effects on the ADGRV1‐WHRN interaction. In contrast, the deletion of PDZ2 from WHRN decreased the ADGRV1‐WHRN interaction by ≈85% but did not significantly affect USH2A‐WHRN coupling (Figure 6b,c).

In contrast to USH2A, which is a single‐transmembrane protein, ADGRV1 is a seven‐transmembrane receptor with a C‐terminal tail plus three ICLs, which might also interact with WHRN. We then employed the FlAsH‐BRET assay to further delineate the mode by which ADGRV1 and WHRN interact. The FlAsH‐BRET method has been successfully used to map conformational changes in GPCRs and arrestins as well as the modes by which ligands and GPCRs interact.^[^ ^17^ ^]^ The FlAsH motif CCPGCC was incorporated into one of the three ICLs of ADGRV1, and NanoLuc (Nluc) was inserted into distinct sites flanking the three PDZ domains of WHRN (denoted L‐Z1, Z1‐L, L‐Z2, Z2‐L, L‐Z3, and Z3‐L, respectively) (Figure 6d and Figure S7b–d, Supporting Information). The interaction between a specific WHRN‐PDZ domain and ADGRV1‐ICL or the assembly of these components is revealed by a BRET signal between the Nluc donor and labeled FlAsH acceptor. Consistently, specific, saturated BRET signals were detected between ADGRV1‐ICL3‐FlAsH and Nluc‐flanked by WHRN‐PDZ2 (L‐Z2 and Z2‐L) as well as between ADGRV1‐ICL2‐FlAsH and Nluc‐flanked by WHRN‐PDZ1 (L‐Z1 and Z1‐L) (Figure 6e). Moreover, the FlAsH‐BRET results also demonstrate the approach of pre‐WHRN‐PDZ3 (L‐Z3) to both ADGRV1‐ICL2 and ADGRV1‐ICL3, indicating the potential involvement of WHRN‐PDZ3 in stabilizing the ALC (Figure 6e).

### 2.8 Mapping the Detailed Interactions between ADGRV1/USH2A and WHRN by NMR

**Figure 7:**
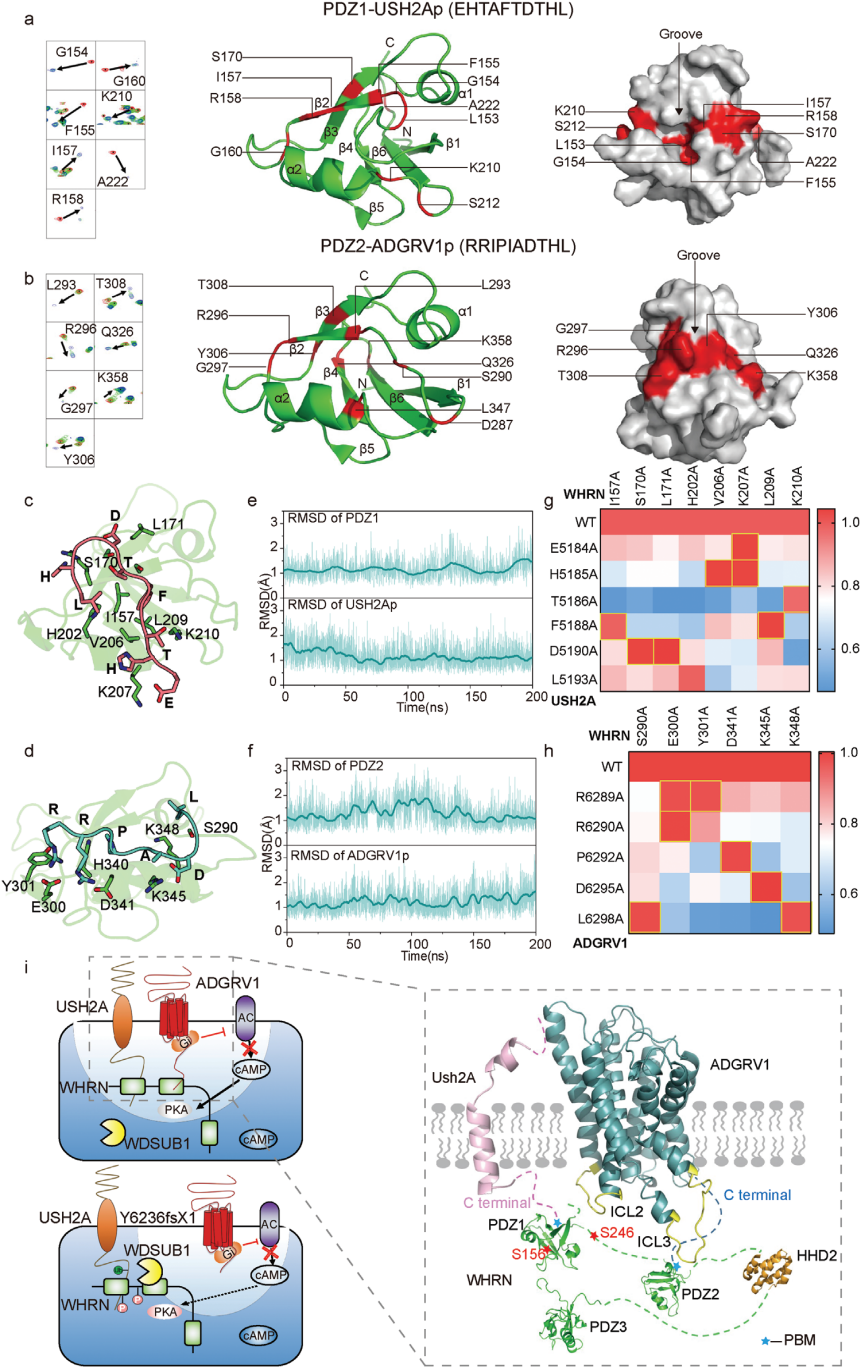
Identification of potential hot spot interactions within ALC ternary complex by NMR, molecular dynamic simulation, and FlAsH‐BRET analysis. a) The cartoon and surface representation of the WHRN‐PDZ1 domain. The structure is derived from the NMR structure of human WHRN‐PDZ1 (PDB ID code: 1UEZ) which has 100% sequence identity with mice WHRN‐PDZ1. The residues with significant chemical shift change (*Δδ* > 0.3 ppm) or resonance intensity reduction (intensity ratio < 20%) upon the addition of USH2Ap are colored in red. b) The cartoon and surface representation of the WHRN‐PDZ2 domain. The structure is derived by Swiss‐model using the template of the NMR structure of human WHRN‐PDZ2 (PDB ID code: 1UF1) which has 97% sequence identity with mice WHRN‐PDZ2. The residues with significant chemical shift change (*Δδ* > 0.2 ppm) or resonance intensity reduction (intensity ratio < 20%) upon the addition of ADGRV1p are colored in red. c) The detailed interactions of C‐terminal residues of USH2A (salmon) with WHRN‐PDZ1 (green). d) The detailed interactions of C‐terminal residues of ADGRV1 (cyan) with WHRN‐PDZ2 (green). e) RMSD analysis of WHRN‐PDZ1/USH2Ap during 200‐ns molecular dynamics simulation trajectories by Gromacs. RMSDs of WHRN‐PDZ1 (upper panel) or peptide residues (lower panel) are shown. The initial modeled complex state after equilibration (0 ns) was used for calculation. f) RMSD analysis of WHRN‐PDZ2/ADGRV1p during 200‐ns molecular dynamics simulation trajectories by Gromacs. RMSDs of WHRN‐PDZ2 (upper panel) or peptide residues (lower panel) are shown. The initial modeled complex state after equilibration (0 ns) was used for calculation. g) Pairing of WHRN mutants with USH2A (WT and mutants) through alanine scanning and FlAsH‐BRET assay. The saturation BRET value between a given WHRN mutant and the WT USH2A was used as the reference (normalized to 1) and the saturation BRET value between the same WHRN mutant and the other USH2A mutant was normalized to the above value. The USH2A mutants that did not show significantly decreased BRET signal compared to the WT USH2A when interacting with a given WHRN mutant are deduced to be potential hot spot interaction pairs and are circled in yellow (for example, USH2A‐F5188 and WHRN‐I157). Data are correlated to Figure S8c, Supporting Information and are from three independent experiments (*n* = 3). h) Pairing of WHRN mutants with ADGRV1 (WT and mutants) through alanine scanning and FlAsH‐BRET assay. Data are processed as described in Figure 7g. Data are correlated to Figure S8d, Supporting Information and are from three independent experiments (*n* = 3). i) Schematics illustrating the interaction mode of ADGRV1‐WHRN‐USH2A ternary complex. The C‐terminal PBM of USH2A and ADGRV1‐ICL2 prefer to interact with WHRN‐PDZ1 while the ADGRV1 C‐terminus and ICL3 favor engaging with WHRN‐PDZ2 (right). Endogenously phosphorylated WHRN recruits WDSUB1 and promotes the ubiquitination and degradation of USH2A. ADGRV1 inhibits WHRN phosphorylation through compartmental cAMP‐PKA signaling and increases USH2A stability (left upper). In contrast, the Y6236fsX1 showed inferior effects on USH2A ubiquitination compared with the wide‐type ADGRV1, due to its inability to form a functional ALC and to locally dephosphorylate WHRN (left bottom).

A detailed structural model of the first two PDZ domains of human WHRN was recently determined by NMR, which has greatly facilitated structural studies of WHRN.^[^ ^18^ ^]^ According to a series of NMR spectra and with reference to NMR signal assignments for human WHRN, most of the residues of the PDZ domains of mouse WHRN were assigned (Figure S7e, Supporting Information). To determine the residues in the PDZ domains of WHRN involved in the recognition of USH2Ap/ADGRV1p, chemical shift perturbation experiments were performed. Obvious chemical shift changes and resonance intensity reductions for PDZ domain residues were observed in response to peptide stimulation, confirming the direct interaction between USH2Ap and WHRN‐PDZ1 and between ADGRV1p and WHRN‐PDZ2 (**Figure** **7**a,b, and Figure S7e,f, Supporting Information). Residues that exhibited a significant chemical shift change or resonance intensity reduction in PDZ1 include those located at the *β*2 sheet and the loop that connects it to the *β*1 sheet (L153, G154, F155, I157, R158, and G160); S170, localized at the *β*3 sheet; and C‐terminal residues (K210, S212, and A222) (Figure 7a). For PDZ2, residues of the *β*2 and *β*3 sheets (L293, R296, G297, Y306, and T308) and ɑ2 helix (L347 and K358), D287 and S290 showed significant chemical shift changes or resonance intensity reductions (Figure 7b). Most of these residues are located in the area surrounding the *β*2 sheet, which is the typical substrate‐binding site (the so‐called “GLGF motif”) of PDZ domains and forms a groove with the ɑ2 helix for USH2Ap/ADGRV1p binding (Figure 7a,b, and Figure S7g,h, Supporting Information). These results corroborate that the PBMs of both USH2Ap and ADGRV1p bind the corresponding PDZ interaction domains of WHRN via classic interaction modes. In particular, residues K210 and S212, which exhibited significant reductions in resonance intensity, are located at the C‐terminus of WHRN‐PDZ1 (Figure S7g–i, Supporting Information), consistent with a previous report showing that the C‐terminus of WHRN‐PDZ1 plays an important role in USH2Ap binding.^[^ ^18b^ ^]^

Moreover, these two binding modes were further supported by 200‐ns molecular dynamic simulation trajectories by GROMACS, which revealed that eight residues from PDZ1 compose the binding pocket for the PBM of USH2Ap, while six residues of PDZ2 may engage with the PBM of ADGRV1p (Figure 7c–f). To further examine the hotspot interactions between WHRN and USH2A/ADGRV1 predicted by computational simulation, we performed a FlAsH‐BRET assay by inserting Nluc into WHRN (L‐Z2 for ADGRV1, Z1‐L for USH2A) and incorporating the FlAsH motif into either ICL3 (between G6084 and Y6085) of ADGRV1 or the intracellular tail (between L5153 and W5154) of USH2A. Whereas specific, saturated BRET signals were detected upon the interaction of WHRN‐Nluc with ADGRV1‐FlAsH or USH2A‐FlAsH, alanine mutations at potential peptide‐binding residues in PDZ1 or PDZ2 of WHRN significantly reduced the BRET signal (Figure S8a,b, Supporting Information). We next paired the interactions of WHRN with key residues in USH2A/ADGRV1 by comparing the binding of respective USH2A/ADGRV1 mutants with that of the WT receptor for all WHRN alanine mutants for which a significant loss of BRET signal is observed. For a WHRN mutant that displays an impaired BRET response to WT USH2A/ADGRV1 but causes no further significant deterioration of the response to USH2A/ADGRV1 with a selective alanine substitution, the WHRN mutation site and USH2A/ADGRV1 substitution site could be paired to identify a potential hotspot interaction. Based on the above principle, we determined that specific interactions between E5184/H5185^USH2A^ and V206/K207^WHRN^; T5186^USH2A^ and K210^WHRN^; F5188^USH2A^ and I157/L209^WHRN^; and D5190^USH2A^ and S170/L171^WHRN^ are potential hotspot interactions between WHRN‐PDZ1 and USH2A. Moreover, the specific interactions between R6289/R6290^ADGRV1^ and E300/Y301^WHRN^, P6292^ADGRV1^ and D341^WHRN^, D6295^ADGRV1^ and K345^WHRN^, and L6298^ADGRV1^ and S290/K348^WHRN^ are potential hotspot interactions between WHRN‐PDZ2 and ADGRV1 (Figure 7g,h, and Figure S8c,d, Supporting Information). Taken together, these results provide details regarding the interaction modes of ALC components at single‐residue resolution.

Combining the FlAsH‐BRET data with the NMR results, we deduced a potential model for the interaction between components of the ADGRV1/WHRN/USH2A complex. The C‐termini of ADGRV1 and ICL3 is in close proximity to the PDZ2 domain of WHRN, whereas the C‐termini of USH2A and ICL2 of ADGRV1 might interact with the PDZ1 domain of WHRN. The PDZ3 domain of WHRN might contact both ICL2 and ICL3 of ADGRV1 (Figure 7i). Forming extensive interactions with both ADGRV1 and USH2A, WHRN acts as a scaffold protein that regulates the ubiquitination of USH2A by WDSUB1. When phosphorylated at S156 and/or S246, WHRN recruits more WDSUB1, enhancing the ubiquitination of USH2A and resulting in its degradation. ADGRV1, by forming a ternary complex with WHRN and USH2A, inhibits WHRN phosphorylation by compartmentalizing cAMP signaling localized around WHRN and increases USH2A stability. In contrast, ADGRV1 Y6236fsX1 fails to inhibit USH2A ubiquitination due to its inability to form a functional complex and regulate the local phosphorylation of WHRN (Figure 7i).

## 3 Discussion

Ankle links are web‐like mesh structures lying at the base of stereocilia, just above the apical surface of developing mammalian cochlear hair cells.^[^ ^1^, ^19^ ^]^ The ALC is composed of at least two large transmembrane proteins, USH2A and ADGRV1, whose malfunction is known to cause congenital deafness and progressive blindness.^[^ ^2^, ^4^, ^6^, ^20^ ^]^ In addition, the cytosolic PDZ domain‐containing scaffold proteins WHRN and PDZD7 participate in ALC formation.^[^ ^6^, ^10^ ^]^ However, the architecture of ALC is poorly defined at present, and how ALC components signal to each other and dynamically regulate ALC formation is unclear. In the current study, we generated *Adgrv1* mutant mice to mimic the deafness‐associated human mutation *ADGRV1* Y6244fsX1. Further characterization of ADGRV1 Y6236fsX1 mice and cellular experiments revealed that regional cAMP‐PKA signaling axis, WHRN phosphorylation, and USH2A ubiquitination play important roles in the dynamic regulation of ALC. Moreover, the architecture of ALC was preliminarily analyzed by the combined use of ITC, SPR, FlAsH‐BRET, and NMR.

In contrast to *Adgrv1* knockout mice, *Adgrv1* Y6236fsX1 mutant mice express nearly intact ADGRV1, with seven integral transmembrane and extracellular domains. Structurally, the ADGRV1 Y6236fsX1 mutant lacks only the C‐terminal 63 aa, which includes the PBM responsible for interacting with WHRN and PDZD7. Notably, the immunostaining results revealed that ADGRV1 Y6236fsX1 is targeted to the apical surface of hair cells rather than at the base of stereocilia, suggesting that this deafness‐associated mutation affects the localization of ADGRV1 in hair cells. The disease association and relatively small structural change in the ADGRV1 Y6236fsX1 mutant suggest that *Adgrv1* Y6236fsX1 mutant mice could serve as an ideal model to study the signal transduction and functions of ankle links and the related pathophysiological processes.

The results herein show that homozygous *Adgrv1* Y6236fsX1 mice are profoundly deaf, their stereocilia are severely disorganized, and their MET currents are significantly decreased. Detailed characterization of *Adgrv1* Y6236fsX1 mice revealed that the assembly of ALC was disrupted and stereocilia development was altered. In neonatal Y6236fsX1 mutant mice, the OHC stereocilia were significantly disorganized, exhibiting a less‐defined V‐shape and reduced bilateral symmetry. On the other hand, the morphology of IHC stereocilia in neonatal *Y6236fsX1* mutant mice was largely normal. Nevertheless, the results of the FM1‐43FX uptake assay showed that MET function was compromised in both the OHCs and IHCs of neonatal *Adgrv1* Y6236fsX1 mutant mice. Furthermore, the third‐row mechanotransducing stereocilia were degenerated in mature *Y6236fsX1* mutant IHCs, consistent with our hypothesis that IHC stereocilia are also affected by this mutation.

It has long been known that ankle links are transient structures that undergo dynamic changes during hair cell development, but the underlying mechanisms have remained largely unknown. Our present data suggest that in *Adgrv1 Y6236fsX1* mutant mice, not only is ALC disassembled due to the absence of the PBM in the ADGRV1 protein but also the protein level of USH2A is significantly decreased. Our cellular experiments indicate that ADGRV1‐modulated phosphorylation of WHRN regulates the ubiquitination and protein stability of USH2A. The lack of appropriate antibodies prevents us from examining the ubiquitination status of USH2A in *Adgrv1* Y6236fsX1 mutant mice. Nevertheless, the significantly reduced USH2A immunoreactivity and increased WHRN phosphorylation level in the cochlea of *Adgrv1* Y6236fsX1 mutant mice is consistent with our in vitro biochemical data. Therefore, our results provide a previously unappreciated mechanism to explain the dynamic regulation of ALC formation by phosphorylation and ubiquitination.

Importantly, we identified the E3 ligase WDSUB1 that regulates the ubiquitination of USH2A. WDSUB1 is a member of the U‐box ubiquitin ligases, which are characterized by the U‐box domain^[^ ^21^ ^]^ WDSUB1 is found only in animals and contains seven tandem WD40 repeats and a SAM domain in addition to the U‐box domain^[^ ^22^ ^]^ The physiological function of WDSUB1 is unclear. The present data show that WDSUB1 is expressed in auditory hair cells and regulates the ubiquitination of USH2A by binding to WHRN. Furthermore, the phosphorylation of WHRN enhances the interactions between WHRN and WDSUB1 and decreases the stability of USH2A, which is inhibited by compartmental cAMP signaling around WHRN mediated by WT ADGRV1 but not the ADGRV1 Y6236fsX1 mutant. These results suggest that local cAMP signaling and the downstream regional phosphorylation of ALC by PKA might play pivotal roles in controlling ALC assembly and degradation.

Previous studies have suggested that the activity of PDE plays important role in the determination of localized cAMP signaling in the cardiovascular or nervous system.^[^ ^23^ ^]^ In addition to the above‐established mechanism, our present study suggested that a complex of a GPCR encompassing the Gi protein and PDZ domain‐containing proteins, such as WHRN, may also contribute to localized cAMP signaling and normal cochlear hair cell functions. The compartmentalization of cAMP signaling is precisely regulated at the nanometer scale and is critical for accurate cellular responses to external stimuli. Disruption of local cAMP signaling is linked to a variety of diseases, such as cancer, heart failure, and nervous system disorders.^[^ ^24^ ^]^ cAMP compartmentalization is achieved by the orchestration of multiprotein signalosomes located at subcellular nanodomains.^[^ ^25^ ^]^ Accordingly, functional cAMP compartmentalization is usually identified and characterized in cells with a unique shape, such as gut epithelial cells and hippocampal neurons, or within spatially confined domains of a given cell, such as the primary cilium.^[^ ^25^, ^26^ ^]^ However, the potential role of cAMP compartmentalization in the stereocilia of cochlear hair cells has never been explored. Further investigations using in vivo compartmentalized cAMP sensors via knock‐in mice or immunofluorescence studies using specific antibodies targeting phospho‐PKA substrates could help to learn more about ADGRV1‐mediated localized G protein signal transduction in hair cells.

To provide deeper insights into the assembly of the ADGRV1/WHRN/USH2A complex as well as how ADGRV1 controls the phosphorylation of WHRN and the ubiquitination of USH2A, we delineated the ADGRV1/USH2A/WHRN ternary complex by ITC, FlAsH‐BRET, and NMR. ITC and SPR experiments indicated that the C‐terminal tail of ADGRV1 is bound to the PDZ2 domain, whereas the tail of USH2A preferentially interacts with the PDZ1 domain of WHRN. This binding mode was further supported by NMR measurements, and the detailed interactions were further dissected by FlAsH‐BRET analysis. Specific residues responsible for the recognition of USH2A by the PDZ1 domain and the recognition of ADGRV1 by the PDZ2 domain were identified by NMR assignments. The structural characterization provided a preliminary interaction mode of ALCs, in which the C‐terminus and intracellular loops of ADGRV1 form extensive interactions with WHRN‐USH2A. These interactions might stabilize the C‐terminus, TM ends, and ICLs of ADGRV1 in a Gi‐favoring conformational state, which potentially explains the enhanced Gi activity of WT ADGRV1 by WHRN and USH2A. To thoroughly dissect the underlying structural basis of the ALC complex and its downstream signaling, the structures of the ADGRV1‐Gi complex and ADGRV1‐WHRN‐USH2A‐Gi megacomplex are required in future studies, which would provide in‐depth knowledge on the working mechanism of ALCs.

## 4 Conclusion

In conclusion, we have recapitulated Usher syndrome type II associated with ADGRV1 mutation by the generation of A*dgrv1* Y6236fsX1 mutant mice. Using this animal model combined with cellular and biophysical approaches, we have provided mechanistic insights into deafness caused by the ADGRV1 Y6236fsX1 mutation, delineated the architecture of the ALC and interactions between its components, and revealed that the phosphorylation of WHRN and ubiquitination of USH2A by the E3 ligase WDSUB1 play critical roles in the dynamic regulation of ALC. Our results suggested that local cAMP signaling and the downstream regional phosphorylation of ALC components by PKA, followed by the recruitment of WDSUB1, might contribute to the developmental degradation of ankle links, which may be an important event in the arc of assembly of a functional hair cell.

## 5 Experimental Section

### Mice

The Shandong University Institutional Animal Care and Use Committee procedure was followed for all animal research. All animal experiments were approved by the Animal Ethics Committee of Shandong University School of Life Sciences (Permit Number: SYDWLL‐2020‐07). *Adgrv1* Y6236fsX1 mutant mice were generated on a C57BL/6J background using CRISPR/Cas9 technology by Shanghai Biomodel Organism Science & Technology Development Co., Ltd as previously described^[^ ^27^ ^]^ Briefly, genomic DNA sequences in mouse *Adgrv1* exon 89 (5ʹ‐TTCTTCTGGAGGTTATGGCC‐3ʹ and 5ʹ ‐TCATCGGCTATCAGGGACCC‐3ʹ) were chosen as the sgRNA‐targets. The *Cas9* mRNA and sgRNA mixture was microinjected into one‐cell embryos together with a DNA oligo containing the 19‐bp deletion as the homology‐directed repair (HDR) template. After the offspring were born, genomic DNA was prepared and screened using PCR and Sanger sequencing to confirm mutations. The founder mice containing the correct mutation were then backcrossed with C57BL/6J mice to give heterozygous mutant mice of F1 generation. The following primers were used for genotyping: 5ʹ ‐ TCTATTCTTACCTCAATCCCA‐3ʹ and 5ʹ ‐TGTCATGTGTGGGTCTCATA‐3ʹ. *Adgrv1*/del7TM knockout mice were obtained from the Jackson Laboratory (Cat. No. 009379).^[^ ^4b^ ^]^

### Cells

Human Embryonic Kidney 293 (HEK293) cells were obtained from American Type Culture Collection (ATCC, Manassas, VA, USA) and cultured in DMEM with 10% FBS. Transient transfection in the current study was performed with Lipofectamine 2000 (Invitrogen) according to the manufacturer's instructions unless otherwise indicated.

### Constructs

The cDNAs encoding ADGRV1a (aa 4333–6298), ADGRV1‐CTF (aa 5884–6298), WHRN, USH2A (aa 4819–5193), and WDSUB1 were cloned into pcDNA3.1 expression vectors to express Flag‐, Myc‐, or EGFP‐fused proteins. ADGRV1 Y6236fsX1 mutant, phospho‐mimic mutants of WHRN (S37E, S85E, S156E, S246E, S156E/S246E, and S384E), potential ubiquitination site mutants of USH2A (K0, K5057/K5060, K5075, K5145/K5146, and K5176/K5183), and the potential hot spot interaction site mutants of ALC (R6289A, R6290A, P6292A, D6295A, L6298A for ADGRV1; E5184A, H5185A, T5186A, F5188A, D5190A, L5193A for USH2A; I157A, S170A, L171A, H202A, V206A, K207A, L209A, K210A, S290A, E300A, Y301A, D341A, K345A, and K348A for WHRN) were generated using the Quikchange mutagenesis kit (Stratagene). For investigating the interaction mode among ALC components using FlAsH BRET assay, the NanoLuc was fused into specific sites flanking respective PDZ domains of WHRN with a four‐amino‐acid linker (Gly‐Ser‐Ser‐Gly) at both sides. FlAsH sequence (Cys‐Cys‐Pro‐Gly‐Cys‐Cys) was inserted into ECL1 (between H5926 and S5927), ELC2 (between H5998 and T5999), or ECL3 (between G6084 and Y6085) of ADGRV1 or into the intracellular C‐tail of USH2A (between L5153 and W5154). The pcDNA3.1‐FluoSTEP‐ICUE probe was a gift from Prof. Jin Zhang (Addgene plasmid # 181845).^[^ ^16^ ^]^All mutations generated in the present study were verified by DNA sequencing.

### ABR Measurements

Mice were placed on an isothermal pad to keep the body temperature at 37 °C during the experiment. After anesthetization with 8.4 mg pentobarbital per 100 g body weight intraperitoneally, electrodes were inserted subcutaneously at the vertex and pinna as well as near the tail. The stimulus generation, presentation, ABR acquisition, and data management were coordinated using an RZ6 workstation and BioSig software (Tucker Davis Technologies, Inc.). Acoustic stimuli (clicks or pure‐tone bursts) of various sound levels were generated using high‐frequency transducers. At each sound level, 512 responses were sampled and averaged. ABR thresholds were determined for each animal as the lowest sound level at which all ABR waves were detectable.

### DPOAE Measurements

Mice were anesthetized and maintained as described above. Two sine wave tones (f2 = 1.2 × f1) were presented for 1‐s durations at various sound levels. The emitted acoustic signal was then picked up by a microphone and digitized, and the magnitude of the distortion product (2 × f1–f2) was determined. The surrounding noise floor was calculated by averaging adjacent frequency bins around the frequency of the distortion product. DPOAE thresholds were determined as the lowest stimulus sound level at which the emitted signal of the distortion product was at least 5 dB SPL above the noise floor.

### Whole‐Mount Immunostaining

The basilar membrane was dissected out and fixed with 4% paraformaldehyde (PFA) in PBS, followed by permeabilization and blocking with PBT1 (0.1% Triton X‐100, 1% BSA, and 5% heat‐inactivated goat serum in PBS, pH 7.3). Samples were then incubated consequentially with the primary antibody in PBT1 and the secondary antibody in PBT2 (0.1% Triton X‐100 and 0.1% BSA in PBS). After that, samples were incubated with phalloidin in PBS, then mounted in PBS/glycerol (1:1), and imaged with a confocal microscope (LSM 700, Zeiss, Germany). Primary antibodies against PDZD7_C, ADGRV1, WHRN, and USH2A were described previously.^[^ ^10b^ ^]^ WDSUB1 antibody was purchased from Thermo Fisher Scientific (Cat. No. PA5‐30695). Alexa Fluor 488‐donkey anti‐rabbit IgG was purchased from Thermo Fisher Scientific (Cat. No. A21202). TRITC‐conjugated phalloidin was purchased from Sigma‐Aldrich (Cat. No. P1951).

### Scanning Electron Microscopy (SEM)

Mouse temporal bones were fixed with 2.5% glutaraldehyde in 0.1 M phosphate buffer overnight at 4 °C. Cochleae were dissected out of the temporal bone and post‐fixed with 1% osmium tetroxide in 0.1 M phosphate buffer at 4 °C for 2 h. Samples were then dehydrated in ethanol and critically point‐dried using a Leica EM CPD300 (Leica, Germany). After that, samples were mounted and sputter coated with platinum (15 nm) using a Cressington 108 sputter coater (Cressington, United Kingdom). The images were taken with a Quanta250 field‐emission scanning electron microscope (FEI, The Netherlands).

### FM 1–43FX Uptake Experiment

Mouse basilar membrane was dissected and incubated with 3 µM FM 1–43FX (Thermo Fisher, Cat. No. F35355) in PBS for 30 s, followed by fixation with 4% PFA at room temperature for 20 min. The samples were then mounted in PBS‐glycerol (1:1) and imaged with a confocal microscope (LSM 700, Zeiss, Germany).

### Hair Cell Electrophysiology

Hair cells were recorded using a whole‐cell patch‐clamp as previously described^[^ ^28^ ^]^ Briefly, the basilar membrane was dissected in the dissection solution containing (in mM) 141.7 NaCl, 5.36 KCl, 0.1 CaCl_2_, 1 MgCl_2_, 0.5 MgSO_4_, 3.4 L‐glutamine, 10 glucose, and 10 H‐HEPES (pH 7.4), then transferred into a recording chamber with recording solution containing (in mM) 144 NaCl, 0.7 NaH_2_PO_4_, 5.8 KCl, 1.3 CaCl_2_, 0.9 MgCl_2_, 5.6 glucose, and 10 H‐HEPES (pH 7.4). A 40‐Hz sinusoidal wave stimulus was delivered by a 27‐mm‐diameter piezoelectric disc driven by a home‐made piezo amplifier pipette with a tip diameter of 3–5 µm positioned 5–10 µm from the hair bundle to evoke maximum MET currents. The evoked MET currents were recorded using a patch‐clamp amplifier (EPC 10 USB and Patchmaster software, HEKA Elektronik, Lambrecht/Pfalz, Germany) with patch pipettes (4‐6 MΏ) filled with an intracellular solution containing (in mM) 140 CsCl, 1 MgCl_2_, 0.1 EGTA, 2 Mg‐ATP, 0.3 Na‐GTP, and 10 H‐HEPES, pH 7.2).

### Immunoprecipitations and Western Blot

For detecting endogenous ADGRV1, the cochlea was isolated from WT or Y6236fsX1 mice, and the proteins were prepared with lysis buffer as described previously.^[^ ^11^, ^29^ ^]^ For detecting overexpressed proteins, transfected HEK293 cells were starved for 12 h at 37 °C and lysed in ice‐cold lysis buffer (50 mM Tris‐HCl, pH 7.4, 150 mM NaCl, 1 mM NaF, 1% Triton, 2 mM EDTA, 1% NP‐40, 1% protease, and phosphatase inhibitor cocktail). The supernatant was then collected after centrifugation and incubated with anti‐Myc antibody or anti‐Flag M2 antibody‐conjugated agarose at 4 °C overnight. Immunoprecipitated proteins were separated by polyacrylamide gel electrophoresis (PAGE), then transferred to the PVDF membrane. After blocking in PBS containing 5% BSA and 0.1% Tween‐20, the membrane was incubated with primary antibody at 4 °C overnight, followed by incubation with HRP conjugate‐secondary antibody at room temperature for an hour. The signals were detected with the ECL system (Thermo Fisher Scientific). Primary antibodies were as follows: anti‐ADGRV1 (Santa Cruz, Cat. No. sc‐21252, sc‐23738); anti‐WHRN (LSBio, Cat. No. LS‐C497481) anti‐Myc (Cell Signaling Technology, Cat. No. 2276); anti‐Flag (Sigma‐Aldrich, Cat. No. F1804); anti‐HA (Cell Signaling Technology, Cat. No. 3724); anti‐Phospho‐(Ser/Thr) PKA Substrate (Cell Signaling Technology, Cat. No. 9621).

For detecting the protein stability of WHRN or USH2A, transfected HEK293 cells were treated with CHX (10 µg ml^−1^) and harvested at different time points (0, 6, 12, and 18 h) for western blot analysis. For analysis of the ubiquitination of USH2A, HEK293 cells were transfected with Flag‐USH2A, HA‐UB, and other components as indicated in the corresponding figures. 48 h after transfection, the cells were treated with E64D (15 µM) for 6 h before whole‐cell extracts were harvested and immunoprecipitated with the Flag‐antibody‐conjugated beads. The precipitate was subjected to western blot analysis and the ubiquitinated USH2A was detected by HA‐antibody.

### Yeast Two‐Hybrid Screening

Yeast strain AH109 (Clontech) was sequentially transformed with the bait plasmid expressing full‐length WHRN and a cochlear cDNA library in the HybriZAP two‐hybrid vector.^[^ ^30^ ^]^ A total of 1.3 × 10^6^ transformants were screened using *HIS3* as the primary reporter gene with the presence of 2.5 mM of 3‐amino‐1,2,4‐triazole (3‐AT). After further examination by two other reporter genes *ADE2* and *lacZ*, the prey vectors in triple‐positive yeast colonies were recovered, and the sequence of cDNA inserts was determined by Sanger sequencing.

### cAMP Assay

The GloSensor‐based real‐time cAMP assay was performed as previously described.^[^ ^11^, ^17^, ^31^ ^]^ HEK293 cells were transiently co‐transfected with the GloSensor and plasmids encoding different proteins (ADGRV1‐CTF, ADGRV1a, Y6236fsX1, or a combination of ADGRV1/WHRN/USH2A or Y6236fsX1/WHRN/USH2A) or empty pcDNA3.1. 24 h after transfection, cells were distributed into 96‐well microplates at a density of 5 × 10^4^ cells per well. After another 24 h incubation at 37 °C, cells were incubated with serum‐free DMEM medium containing 2% (w/w) GloSensor cAMP substrate (Promega) for 2 h at 37 °C. The cells were stimulated with 10 µM Forskolin before the luminescence of each well was immediately recorded using a multimode plate reader (EnVision, PerkinElmer) for 20 min.

### FRET Measurement

The cAMP dynamics in a global cell or around WHRN were evaluated using FluoSTEP probes as previously described.^[^ ^16^ ^]^ Briefly, the HEK293 cells were transiently co‐transfected with the actin‐FluoSTEP‐ICUE probe, WHRN, USH2A, and ADGRV1 (WT or Y6236fsX1 or empty pcDNA3.1), or with WHRN‐FluoSTEP‐ICUE probe, USH2A, and ADGRV1 (WT or Y6236fsX1 or empty pcDNA3.1) in 35‐mm dishes. After 48 h, the transfected cells were washed twice with HBSS solution and imaged in a dark environment at room temperature. Fluorescence imaging was performed on a Zeiss LSM 880 laser scanning microscope (Carl Zeiss) equipped with a plan apochromat 63 × /1.4‐numerical‐aperture oil objective, main dichroic beam splitter 488/554/633, and a GaAsP detector. Fluorescence was excited using the 488 nm Argon laser and collected in the ranges: 493–598 nm (GFP channel) and 638–747 nm (G‐R channel). For fluorescence correction, the background fluorescence of a cell‐free region was subtracted from the fluorescence of the target cells. The exposure time was 500 ms and images were acquired every 30 s. After 5 min to establish the baseline emission ratio, 50 µM Fsk (in the presence of 100 µM IBMX) was added and the images were acquired for another 15 min. All data were analyzed using ZEN black software and the emission ratio was calculated according to the following equation: EM ratio (G/R) = (GFP EM_target_ − GFP EM_Background_)/(G‐R EM_target_ − G‐R EM_Background_). Data were plotted by normalizing the emission ratio of a given time point after Fsk treatment (*R*) to the baseline emission ratio (*R*_0_). For data quantification, the summary panels of Figure 4c and Figure S4h, Supporting Information were plotted as the normalized‐to‐max emission ratio change (*△R*/*△R_max_*), which was calculated as (*R* − *R*_0_) / (*R_max_* −*R*_0_), where *R* and *R*_0_ are defined as above and *R*_max_ is the maximum ratio value recorded in control cells (without ADGRV1 expression) after Fsk/IBMX stimulation.

### ITC Assay

The purified protein (50 uM) was dialyzed into a buffer containing 20 mM Tris, 200 mM NaCl, and 2 mM EDTA, pH 7.4. The peptide (1 mM) was dissolved in the above buffer. The ITC experiment was performed on an ITC200 (GE Healthcare) instrument. The experimental data were processed using MicroCal LLC software.

### SPR Assay

Interaction between ADGRV1p/USH2Ap and PDZ domains of WHRN were analyzed using the BiaCore T200 system. WHRN‐PDZ1 or WHRN‐PDZ2 protein was immobilized on a CM5 chip, and ADGRV1p or USH2Ap peptide diluted in running buffer (10 mM HEPES, 150 mM NaCl, 0.05% Surfactant P20, 0.01% DDM, and 0.002% CHS, pH 7.4) was injected as an analyte with the concentrations in serial dilutions for 120 s as the period of association. Subsequently, the running buffer was alternatively perfused over the chip to allow the bound ADGRV1p or USH2Ap peptide to undergo another 120‐s period of disassociation. The altered response units (ΔRUs) were recorded and the KD of the peptide‐PDZ interaction was calculated accordingly.

### Molecular Dynamic Simulation of ADGRV1/USH2A‐WHRN Interaction

The CHARMM36m force field was used for WHRN‐PDZs, ADGRV1p, USH2Ap lipids, ions, and the TIP3P model water molecules. 0.15 M NaCl was added to balance the charge of the system using the Monte Carlo method. Next, the four systems were solvated into a periodic TIP3P water box with a size of ≈70 × 70 × 100 Å. The box type was set as hexagonal. The systems were first subjected to energy minimization for 10 000 steps with Gromacs2019.5, of which the first 5000 steps were performed using the steepest descent method and the remaining 5000 steps using the conjugated gradient method. The four systems were heated from 0 to 310 K in the NVT ensemble for over 1000 ps. Following this, production simulations were run at 1 atm in the NPT ensemble for over 1000 ps with 10.0 kcal mol^−1^ Å^−2^ harmonic restraints. The NVT ensemble, NPT ensemble, and the 200 ns MD production with a time step of 2 fs were performed by Gromacs2019.5. The particle mesh Ewald (PME) method was used to calculate electrostatic interactions with a cut‐off of 12 Å. The SHAKE algorithm was used for constraining the bonds involving hydrogen atoms during each integration time step of 2 fs.

### FlAsH‐BRET Assay

The FlAsH‐BRET assay was performed as previously described.^[^ ^17b,d^ ^]^ HEK293 cells were transiently transfected to express a fixed amount of WHRN‐Nluc and an increasing concentration of ADGRV1‐FlAsH or USH2A‐FlAsH (or these FlAsH‐tagged acceptor‐based mutants) and incubated for 48 h at 37 °C. The transfected cells were labeled with 2.5 µM FlAsH‐EDT2 for 40 min at 37 °C. After washing twice with PBS, the cells were resuspended and seeded into a 96‐well plate at a density of 5 × 10^4^ cells per well. After adding luciferase substrate coelenterazine‐h at a final concentration of 5 µM, the light intensity emitted by the fluorescence acceptor (FlAsH, 530 nm) and that emitted by fluorescence donor (Nluc, 485 nm) were recorded using a Mithras LB 940 multi‐mode microplate reader. The BRET signal was calculated as the ratio of the Em530 over Em485. The net BRET was determined by subtracting the BRET signal obtained in the cells transfected only with WHRN‐Nluc in the respective experiment.

### Cell‐Surface ELISA Assay

To evaluate the ADGRV1 (WT or FlAsH mutants) expression on the cell surface, HEK293 cells were transiently transfected with varying concentrations of plasmid encoding target receptors in six‐well plates. After 24 h, cells were seeded into a 96‐well plate at a density of 4 × 10^4^ cells per well and incubated at 37 °C in a 5% CO_2_ atmosphere for another 24 h. The cells were then fixed in DPBS containing 4% (w/v) paraformaldehyde for 10 min. The cells were blocked with 5% (w/v) BSA for at least 1.5 h at 37 °C and then incubated with the anti‐FLAG primary antibody overnight at 4 °C. After washing, the cells were incubated with a secondary horseradish peroxidase‐conjugated goat anti‐mouse antibody for 1 h at room temperature. 200 µl TMB (3,3′,5,5′‐tetramethylbenzidine) solution was added into each well to induce the color reaction, which was quenched by adding an equal volume of 0.25 M HCl solution. The absorbance at 450 nm was recorded using a TECAN luminescence counter (Infinite M200 Pro NanoQuant) to determine the relative repression levels of ADGRV1 and the mutants.

### Mass Spectrometry Analysis

HEK293 cells transfected with plasmid encoding Flag‐WHRN were stimulated with 10 µM FSK or control vehicle for 30 min in the presence of IBMX (0.5 mM) before the whole‐cell extracts were immunoprecipitated with anti‐Flag M2 antibody‐conjugated agarose. The bound proteins were eluted with 20 mM HEPES buffer containing 0.2 mg ml^−1^ FLAG peptide, then subjected to trypsin digestion overnight. The resulting peptides were desalted and dried in a speed vac, then separated by HPLC (Easy nLC 1000‐Thermo Scientific) and injected into a mass spectrometer (LTQ Orbitrap Elite‐Thermo Scientific). Typical MS conditions were a spray voltage of 2 kV and a capillary temperature of 275 °C. Survey full‐scan MS spectra were acquired in the orbitrap (m/z 300−1600) with a resolution of 240 000. Precursor ions were fragmented by collision‐induced dissociation and MS2 scans were acquired in the linear ion trap using the Top10 method. The acquired MS data files were processed and searched against the UniProt mouse proteome database (downloaded Feb 2020) using Proteome Discoverer 1.4 (Thermo Scientific).

### NMR Spectroscopy

The 0.7 mM purified ^13^C, ^15^N‐labeled proteins (PDZ1 and PDZ2) were dialyzed into phosphate buffer containing 25 mM NaH_2_PO_4_, 100 mM NaCl, and 2 mM EDTA, pH 6.8. All NMR data were collected at 293 K on a Bruker DMX600 spectrometer. For backbone resonance assignments, ^1^H‐^15^N HSQC, CBCANH, and CBCA(CO)NH spectra were recorded. NMR data were processed with NMRpipe and Sparky3 software. ^15^N‐labeled protein was titrated with an increasing concentration of peptide to different molar ratios (PDZ1/USH2Ap = 1:0, 1:0.5, 1:1, 1:2, 1:3, 1:4, PDZ2/ ADGRV1p = 1:0, 1:0.5, 1:1, 1:2, 1:4, 1:5). Chemical shift perturbations (CSP) were calculated as the weighted average (^1^H, ^15^N) chemical shift by the equation *Δδ*avg = ((Δ*δ*H)^2^ + (0.159Δ*δ*N)^2^)^1/2^. The signal intensity change of each amino acid was processed with Sparky3 software.

### Statistical Analysis

All data were represented as mean ± SEM with the numbers of experiments or mice indicated in the Figure legends. Unpaired two‐tailed Student *t‐*test was used for the comparison between two groups and one‐way or two‐way ANOVA followed by Dunnett's post‐hoc test was used for comparisons among multiple groups. Statistical analyses were performed using GraphPad Prism 8 software. No methods were used to determine whether the data met the assumptions of statistical approach and no power analysis was performed to determine the sample size. The sample size was based on experience in previous studies from the lab using animal models. Statistical values are represented as significant when *p‐*values were below 0.05. For all tests, *, **, and *** stand for *p* < 0.05, *p* < 0.01, and *p* < 0.001, respectively.

## Conflict of Interest

The authors declare no conflict of interest.

## Author Contributions

Y.G., H.‐B.D., Z.Y., Y.‐Z.W., R.R., and W.‐W.L. contributed equally to this work. Z.‐G.X. and J.‐P.S. conceived and initiated the project. Z.‐G.X., J.‐P.S., X.‐M.T., and R.‐J.C. supervised the overall project execution. H.‐B.D., R.R., W.‐W.L., Y.‐X.S., and Y.‐F.W. characterized *Adgrv1* Y6236fsX1 mice and performed behavioral, SEM, immunostaining, and FM1‐43FX uptake experiments. H.‐B.D. cloned the cDNAs encoding ALC components and WDSUB1 from the mouse's inner ear. J.L. and W.X. performed MET measurements. J.Y. and W.‐W.L. provided essential reagents and participated in data analysis. J.‐P.S. designed all the cell‐based functional assays for characterizing the ALC interaction mode. Z.Y., Y.G., and X.‐X.Z. generated all constructs and mutants for the cell‐based functional assays. Z.Y. and Y.G. performed cell surface ELISA assay, Western blotting, co‐immunoprecipitation, cAMP accumulation assay, and FRET imaging. H.‐B.D. and N.‐N.L. performed yeast two‐hybrid screening. J.‐P.S. designed the constructs for the FlAsH‐BRET assay. Z.Y. and Y.G. generated the constructs and performed the FlAsH‐BRET analysis. C.Z. performed MD stimulation. Z.‐Y.Y. performed the SPR experiment. Y.‐Z.W. and F.‐Z.S. performed ITC and NMR experiments under the guidance and supervision of X.‐M.T. Z.Y., Y.G., H.‐B.D. R.R., and Y.‐Z.W. participated in the preparation of figures. J.‐P.S., Z.‐G.X., X.‐M.T., and R.‐J.C. participated in data analysis and interpretation. J.‐P.S., Z.‐G.X., and X.‐M.T. wrote the manuscript. All the authors have read and commented on the manuscript.

## Supporting information

Supporting information

## Data Availability

The data that support the findings of this study are available from the corresponding author upon reasonable request.
