# Supplementary material for: Deafness‐Associated ADGRV1 Mutation Impairs USH2A Stability through Improper Phosphorylation of WHRN and WDSUB1 Recruitment
Source: Adv Sci (Weinh). 2023 Apr 17;10(16):2205993. doi: 10.1002/advs.202205993 (PMC10238197; doi:10.1002/advs.202205993)
Supplement: Supplementary file 1 — Supporting information [file ADVS-10-2205993-s001.pdf]

ADVANCED SCIENCE
Open Access

Supporting Information

for *Adv. Sci.*, DOI 10.1002/advs.202205993

Deafness-Associated ADGRV1 Mutation Impairs USH2A Stability through Improper Phosphorylation of WHRN and WDSUB1 Recruitment

Ying Guan, Hai-Bo Du, Zhao Yang, Yu-Zhu Wang, Rui Ren, Wen-Wen Liu, Chao Zhang, Jia-Hai Zhang, Wen-Tao An, Na-Na Li, Xiao-Xue Zeng, Jie Li, Yi-Xiao Sun, Yan-Fei Wang, Fan Yang, Jun Yang, Wei Xiong, Xiao Yu, Ren-Jie Chai, Xiao-Ming Tu, Jin-Peng Sun* and Zhi-Gang Xu**

Deafness-associated ADGRV1 mutation impairs USH2A stability through improper phosphorylation of WHRN and WDSUB1 recruitment

Ying Guan#, Hai-Bo Du#, Zhao Yang#, Yu-Zhu Wang#, Rui Ren#, Wen-Wen Liu#, Chao Zhang, Jia-Hai Zhang, Wen-Tao An, Na-Na Li, Xiao-Xue Zeng, Jie Li, Yi-Xiao Sun, Yan-Fei Wang, Fan Yang, Jun Yang, Wei Xiong, Xiao Yu, Ren-Jie Chai, Xiao-Ming Tu, Jin-Peng Sun*, and Zhi-Gang Xu**

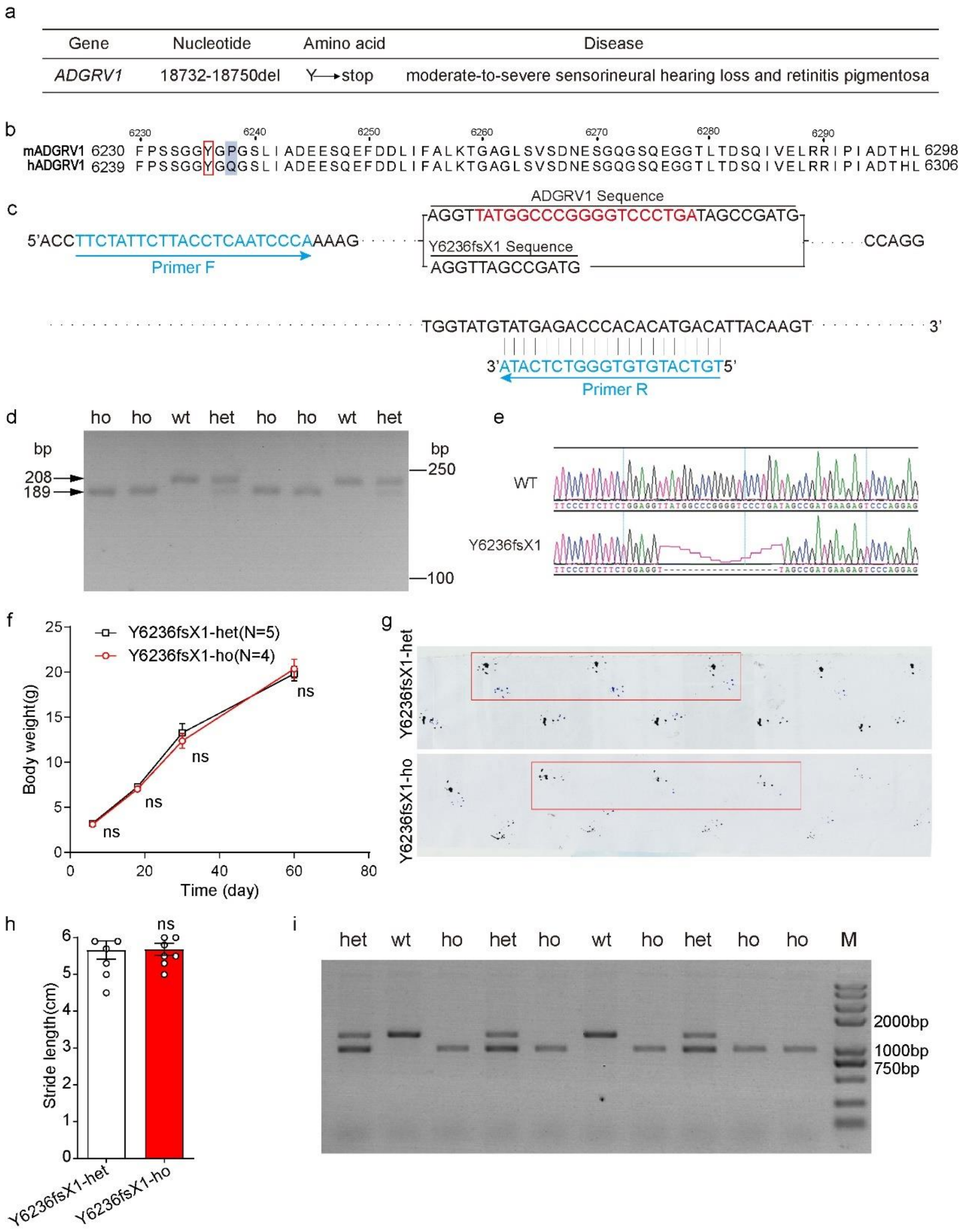

Figure s1. Generation and characterization of *Adgrv1* Y6236fsX1 mice.

a) Association of *ADGRV1* Y6244fsX1 mutation with human sensorineural hearing loss

and retinitis pigmentosa.

b) Sequence alignment of the intracellular cytoplasmic terminus between human and mouse ADGRV1. Y6244 in human ADGRV1 and the equivalent Y6236 in mouse ADGRV1 are highlighted with red box.

c) Schematic representation of the primers used in the genotyping of the *Adgrv1* Y6236fsX1 mice. The 19 bp sequence deleted in the *Adgrv1* Y6236fsX1 mice is indicated in red.

d) A representative genotyping PCR result showing amplified fragments derived from *Adgrv1* Y6236fsX1 mice and their wild type littermates.

e) Sanger sequencing confirmed a 19bp deletion in *Adgrv1* mRNA of *Adgrv1* Y6236fsX1 mice.

f) Body weight changes of heterozygous and homozygous *Adgrv1* Y6236fsX1 mice (n=5 for heterozygous mice and n=4 for homozygous mice).

g-h) Representative images (g) and stride length analysis (h) of footprint test of heterozygous and homozygous *Adgrv1* Y6236fsX1 mice. The footprints of forelimbs and hindlimbs were labelled by blue and black inks, respectively (n=6).

i) A representative genotyping PCR result showing amplified fragments derived from *Adgrv1*/del7TM mice and their wild type littermates.

Data information: f, h) ns, no significant difference; heterozygous *Adgrv1* Y6236fsX1 mice compared with homozygous mice. The bars indicate the mean ± SEM values. Data were statistically analyzed using two-way ANOVA with Dunnett's post hoc test (f) or student's *t* test (h).

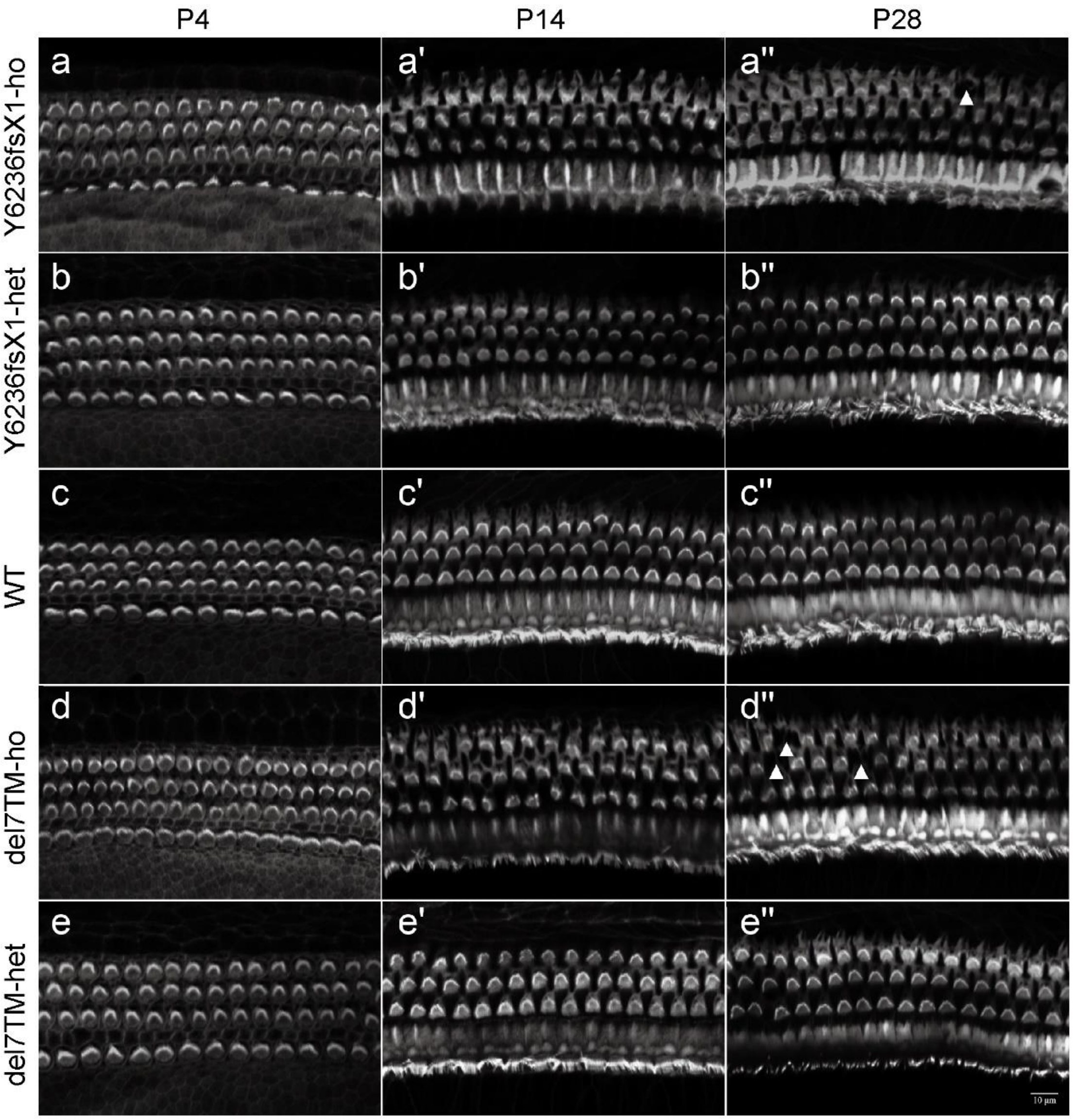

Figure s2. ***Adgrv1*** **Y6236fsX1 mutation leads to stereocilia disorganization.**

a-e) Whole-mount phalloidin-immunostaining of the cochlear hair cell stereocilia in homozygous *Adgrv1 Y6236fsX1* mutant mice (a-a”), heterozygous *Adgrv1 Y6236fsX1* mutant mice (b-b”), wild-type mice (c-c”), homozygous *Adgrv1*/del7TM mice (d-d”), and heterozygous *Adgrv1*/del7TM mice (e-e”) at different ages as indicated. Scale bar: 10 μm.

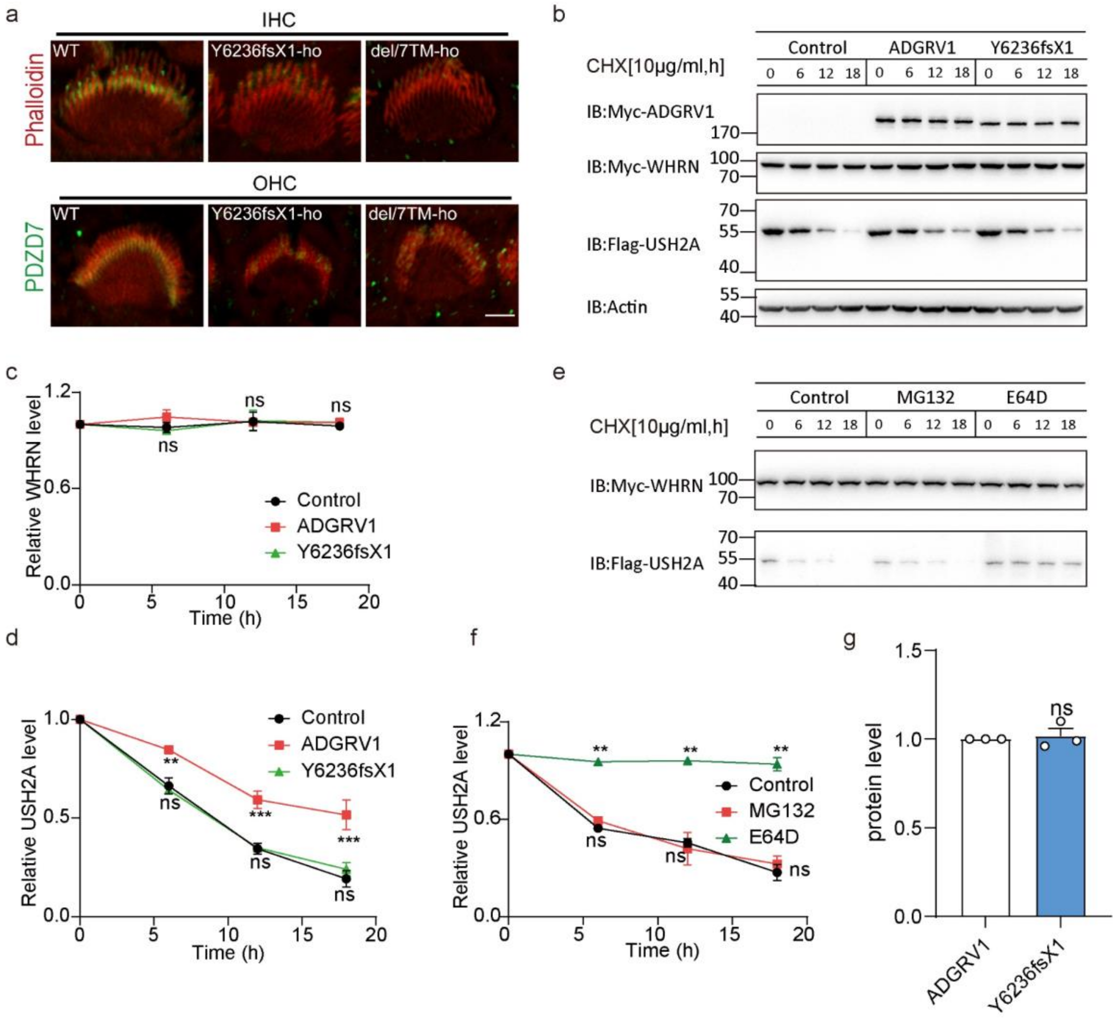

Figure s3. ***Adgrv1*** **Y6236fsX1 mutation decreases USH2A stability** ***in vitro*****.**

a) Whole-mount immunostaining showing the localization of ALC components PDZD7 in the stereocilia of IHC and OHC from P5 mice of different genotypes as indicated. TRITC-phalloidin was used to visualize the F-actin core of stereocilia. All images were taken from the middle turn of the basilar membrane using a confocal microscope. Scale bar: 2 μm. Representative images from three independent experiments were shown.

b) Representative blotting showing the time-dependent protein levels of WHRN and USH2A in HEK293 cells co-transfected with plasmids encoding Flag-USH2A, Myc-WHRN, and ADGRV1 (WT or Y6236fsX1 mutant) in the presence of 10 μg/ml cycloheximide (CHX).

c-d) Relative WHRN (c) and USH2A (d) levels in HEK293 cells co-transfected with plasmids encoding Flag-USH2A, Myc-WHRN, and ADGRV1 (WT or Y6236fsX1 mutant). Data are correlated with Fig. s3b and the band intensity of WHRN or USH2A in the control HEK293 cells transfected with Myc-WHRN and Flag-USH2A (without ADGRV1) before CHX

treatment was used as the reference (normalized to 1). Data are from three independent experiments (n=3).

e) Representative blotting showing the effects of MG132 or E64D on the time-dependent protein level of USH2A in HEK293 cells that were co-transfected with plasmids encoding Flag-USH2A and Myc-WHRN in the presence of 10 μg/ml cycloheximide (CHX). The transfected cells were treated with 20 μM MG132 or 15 μM E64D.

f) Relative USH2A levels in Flag-USH2A and Myc-WHRN overexpressed HEK293 cells that are pretreated with MG132 or E64D. Data are correlated with Fig. s3e and the band intensity of USH2A in the control group transfected with Myc-WHRN and Flag-USH2A before CHX treatment was used as the reference (normalized to 1). Data are from three independent experiments (n=3).

g) Relative WT ADGRV1 and Y6236fsX1 mutant protein levels in HEK293 cells transfected with Myc-WHRN, Flag-USH2A, Myc-ADGRV1(WT or Y6236fsX1) and HA-UB. Data are correlated with Fig. 3e and are from three independent experiments (n=3).

c-d) **$P < 0.01$; ***$P < 0.001$; ns, no significant difference; HEK293 cells transfected with ADGRV1/WHRN/USH2A or Y6236fsX1/WHRN/USH2A compared with those transfected with WHRN/USH2A. f) **$P < 0.01$; ns, no significant difference; HEK293 cells treated with MG132 or E64D compared with those treated with control vehicle. g) ns, no significant difference; HEK293 cells transfected with ADGRV1/WHRN/USH2A/HA-UB compared with those transfected with Y6236fsX1/WHRN/USH2A/HA-UB. The bars indicate the mean ± SEM values. Data were statistically analyzed using two-way (c-d, f) with Dunnett's post hoc test, or student's *t* test (g).

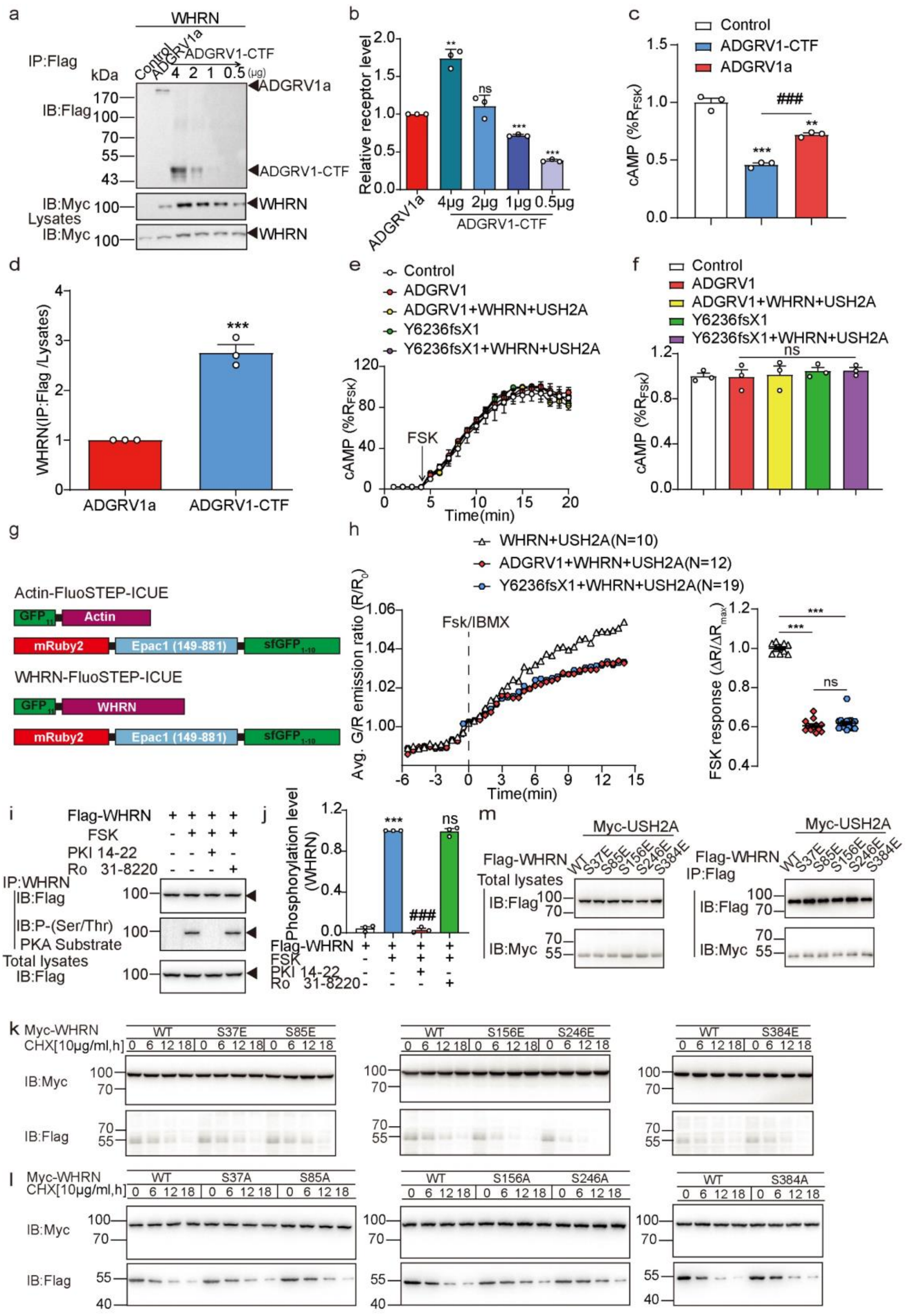

Figure s4. Phosphorylation state of WHRN mediated by cAMP-PKA axis regulates USH2A stability.

a) Representative blotting showing the co-immunoprecipitation of WHRN with ADGRV1 in HEK293 cells transfected with Myc-WHRN and Flag-ADGRV1a or with Myc-WHRN and

varying amounts of Flag-ADGRV1-CTF.

b) Relative ADGRV1 expression levels in HEK293 cells transfected with Flag-ADGRV1a or with varying amounts of Flag-ADGRV1-CTF. Data are correlated to Fig. s4a and are from three independent experiments (n=3). HEK293 cells transfected with 4 μg plasmids encoding Flag-ADGRV1a showed similar expression level of the receptor to those transfected with 2 μg plasmids encoding Flag-ADGRV1-CTF, and were used for subsequent cAMP assay (Fig. s4c) and WHRN immunoprecipitation analysis (Fig. s4d).

c) Effects of Flag-ADGRV1a or Flag-ADGRV1-CTF on the FSK-induced cAMP accumulation in HEK293 cells. Data were normalized to the maximal cAMP response in control cells that were transfected with empty vector pcDNA3.1 and are from three independent experiments (n=3).

d) The co-immunoprecipitated Myc-WHRN levels with Flag-ADGRV1a or Flag-ADGRV1-CTF when these two isoforms of ADGRV1 were expressed at similar level. Data are correlated with Fig. s4a and are from three independent experiments (n=3).

e-f) Effect of PTX pretreatment on the FSK-stimulated cAMP levels in HEK293 cells transfected with ADGRV1 or Y6236fsX1 in the absence or presence of WHRN and USH2A. Both the time-course of cAMP production (e) and quantitative analysis (f) were shown. Data are from three independent experiments (n=3).

g) Schematic representation of the Actin-FluoSTEP-ICUE and WHRN-FluoSTEP-ICUE probes.

h) Left: Time course of cAMP production indicated by average G/R emission ratio in HEK293 cells expressing Actin-FluoSTEP-ICUE probe, WHRN, USH2A and ADGRV1 (WT or Y6326fsX1 mutant or empty vector pcDNA3.1) following stimulation with 50 μM Fsk and 100 μM IBMX. Right: Summary of emission ratio change (△R/△Rmax) for Actin-FluoSTEP-ICUE (△R/△Rmax; see Experimental section). Data are from 10-19 cells from three independent experiments.

i-j) Representative blotting (i) and quantitative analysis (j) of the effects of different kinase inhibitors on FSK-stimulated WHRN phosphorylation. Data are from three independent experiments (n=3).

k-l) Representative blotting showing the time-dependent protein levels of WHRN and USH2A

in HEK293 cells co-transfected with Flag-USH2A and Myc-WHRN (WT or mutants) in the presence of 10 μg/ml cycloheximide (CHX).

m) Representative blotting showing the Myc-USH2A coimmunoprecipitated with Flag-tagged WHRN (WT or phospho-mimic mutants) in HEK293 cells. Data are correlated with Fig. 4n.

b) **P < 0.01; ***P < 0.001; ns, no significant difference; HEK293 cells transfected with Flag-ADGRV1-CTF compared with those transfected with Flag-ADGRV1a. c) **P < 0.01; ***P < 0.001; HEK293 cells transfected with Flag-ADGRV1-CTF or Flag-ADGRV1a compared with those transfected with pcDNA3.1; $^{###}$P < 0.001; HEK293 cells transfected with 2 μg plasmids encoding Flag-ADGRV1-CTF compared with those transfected with 4 μg plasmids Flag-ADGRV1a. d) ***P < 0.001; HEK293 cells transfected with 2 μg plasmids encoding Flag-ADGRV1-CTF compared with those transfected with 4 μg plasmids Flag-ADGRV1a. f) ns, no significant difference; HEK293 cells transfected with ALC components compared with those transfected with the pcDNA3.1. h) ***P < 0.001; HEK293 cells transfected with ADGRV1 (WT or mutant) compared with those transfected with empty vector. ns no significant difference; HEK293 cells transfected with Y6236fsX1 compared with those transfected with WT ADGRV1. j) ***P < 0.001; HEK293 cells treated with FSK compared with those treated with control vehicle. $^{###}$P < 0.001; ns, no significant difference; HEK293 cells pretreated with kinase inhibitors compared with those pretreated with control vehicle. The bars indicate the mean ± SEM values. Data were statistically analyzed using one-way ANOVA with Dunnett's post hoc test (b-c, f, h, j) or student's *t* test (d).

a

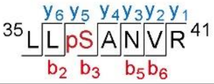

Ion Type	m/z	Sequence	Ion Type	m/z
b_2^{+}	227.18	L36	y_6^{+}	739.35
b_3^{2+}	197.59	pS37	y_5^{+}	626.26
		A38	y_4^{+}	459.26
b_5^{+}	579.25	N39	y_3^{+}	388.23

b

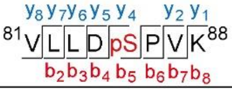

Ion Type	m/z	Sequence	Ion Type	m/z
b_3^{+}	326.24	L83	y_7^{+}	894.44
b_4^{+}	441.27	D84	y_6^{+}	781.36
b_5^{+}	608.27	pS85	y_5^{+}	666.33
b_6^{+}	705.32	P86	y_4^{+}	499.34

c

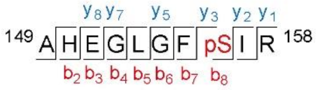

Ion Type	m/z	Sequence	Ion Type	m/z
b_6^{+}	565.11	G154	y_5^{+}	659.35
b_7^{+}	712.50	F155		
b_8^{+}	879.56	pS156	y_3^{+}	455.34
		I157	y_2^{+}	288.45

d

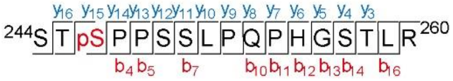

Ion Type	m/z	Sequence	Ion Type	m/z
		S244		
		T245	y_{16}^{2+}	871.41
		pS246	y_{15}^{2+}	820.89
b_4^{+}	533.10	P247	y_{14}^{+}	1473.78

e

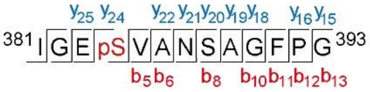

Ion Type	m/z	Sequence	Ion Type	m/z
		E383	y_{25}^{3+}	898.06
		pS384	y_{24}^{2+}	1282.06
b_5^{2+}	283.61	V385		
b_6^{+}	637.26	A386	y_{22}^{2+}	1149.03

Figure s5. Mass spectrometry analysis revealing potential phosphorylation sites in WHRN.

a-e) Mass spectrometry data of immunoprecipitated WHRN from FSK-stimulated HEK293 cells transfected with Myc-WHRN revealed potential PKA phosphorylation sites, including S37 (a), S85 (b), S156 (c), S246 (d), and S384 (e).

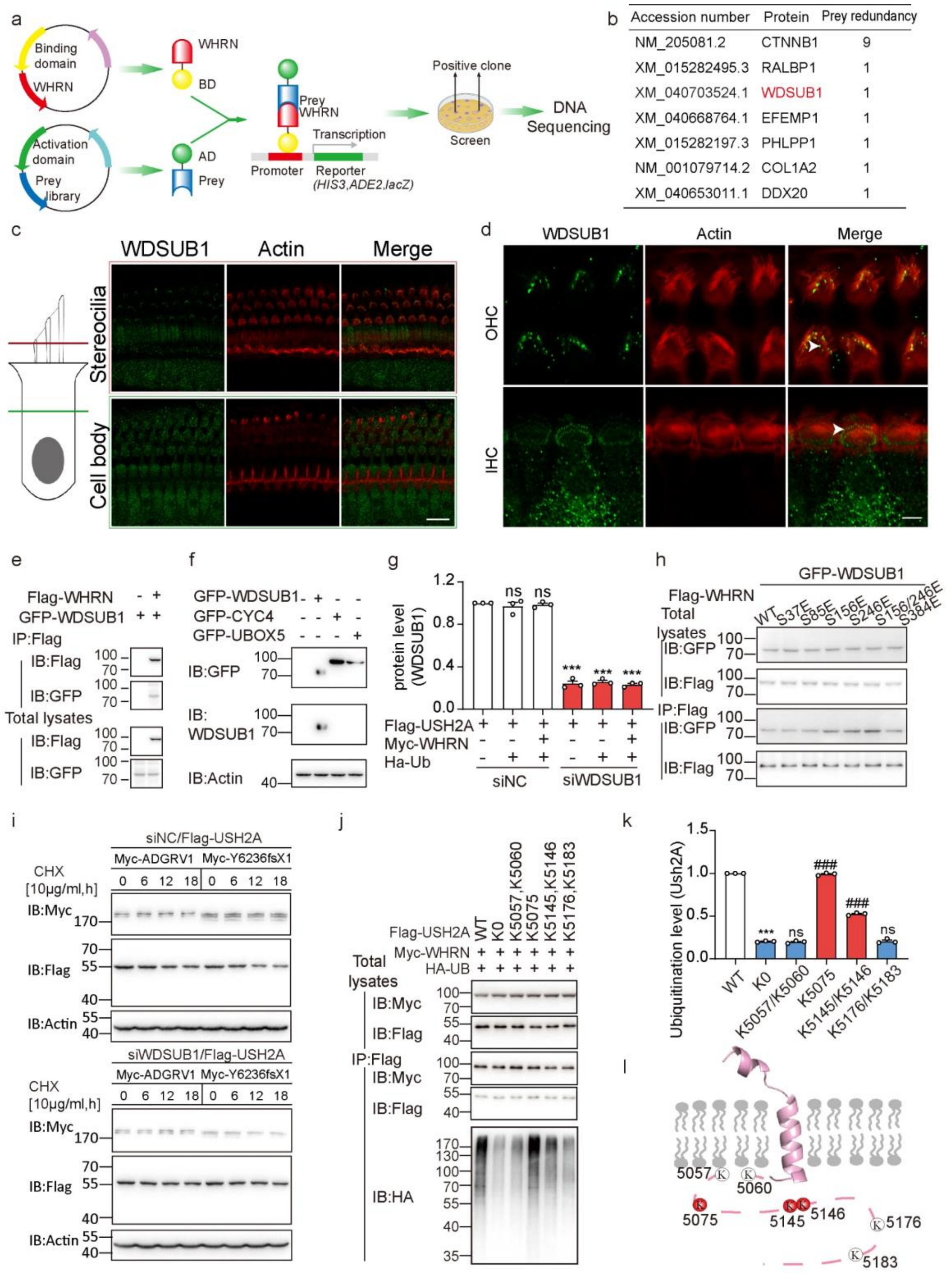

Figure s6. WDSUB1 induces USH2A ubiquitination in WHRN-dependent manner.

a) Schematic diagram of the yeast two-hybrid screening of a cochlear cDNA library using full-length WHRN as a bait.

b) Potential WHRN-binding partners identified from yeast two-hybrid screening.

c) Whole-mount immunostaining showing WDSUB1 (green) and actin (magenta) in the auditory hair cells of WT mice at the stereocilia layer or cell body layer. Scale bar: 10

μm. Representative images from three independent experiments were shown.

d) Whole-mount immunostaining showing WDSUB1 (green) and actin (magenta) in the auditory OHC and IHC of WT mice. Scale bar: 4 μm. Representative images from three independent experiments were shown.

e) Representative blotting showing the coimmunoprecipitation of GFP-WDSUB1 with Flag-WHRN in HEK293 cells.

f) The specificity of the WDSUB1 antibody was verified by western blotting analysis of the GFP-tagged U-box ubiquitin ligase family member in HEK293 cells. The GFP antibody was used as the control.

g) The knockdown efficiency of WDSUB1 siRNA was verified by evaluating the WDSUB1 levels in HEK293 cells transfected with different ACL components with the treatment of control or WDSUB1 siRNA. Data are correlated with Fig. 5e.

h) Representative blotting of the GFP-WSDUB1 coimmunoprecipitated with Flag-WHRN (WT or phospho-mimic mutants) in HEK293 cells. Data are correlated with Fig. 5g and are from three independent experiments (n=3).

i) Representative blotting of the stability of USH2A in ADGRV1 (WT or Y6236fsX1) overexpressed HEK293 cells that were treated with control or WDSUB1 siRNA. Data are correlated with Fig. 5h and are from three independent experiments (n=3).

j-k) Representative blotting (j) and quantitative analysis (k) of the ubiquitination levels of USH2A in HEK293 cells transfected with Myc-WHRN, HA-UB, and Flag-USH2A (WT or mutants with specific lysine substituted with arginine). Data were normalized to the ubiquitination level of USH2A in HEK293 cells transfected with Myc-WHRN, HA-UB, and WT Flag-USH2A. Data are from three independent experiments (n=3).

l) Schematic representation showing the potential unbiquitination sites at the cytoplasmic tail of USH2A.

g) ns, no significant difference; HEK293 cells transfected with USH2A and WHRN/UB compared with those transfected only with USH2A. ***P < 0.001; HEK293 cells treated with siWDSUB1 compared with those treated with control siRNA. k) ***P < 0.001; HEK293 cells transfected with Myc-WHRN, HA-UB, and Flag-USH2A K0 mutant compared with those

transfected with Myc-WHRN, HA-UB, and WT Flag-USH2A. $^{\#\#\#}P < 0.001$; ns, no significant difference; HEK293 cells transfected with Myc-WHRN, HA-UB, and USH2A K0-based mutants compared with those transfected with Myc-WHRN, HA-UB, and Flag-USH2A K0. The bars indicate the mean ± SEM values. Data were statistically analyzed using one-way ANOVA with Dunnett’s post hoc test.

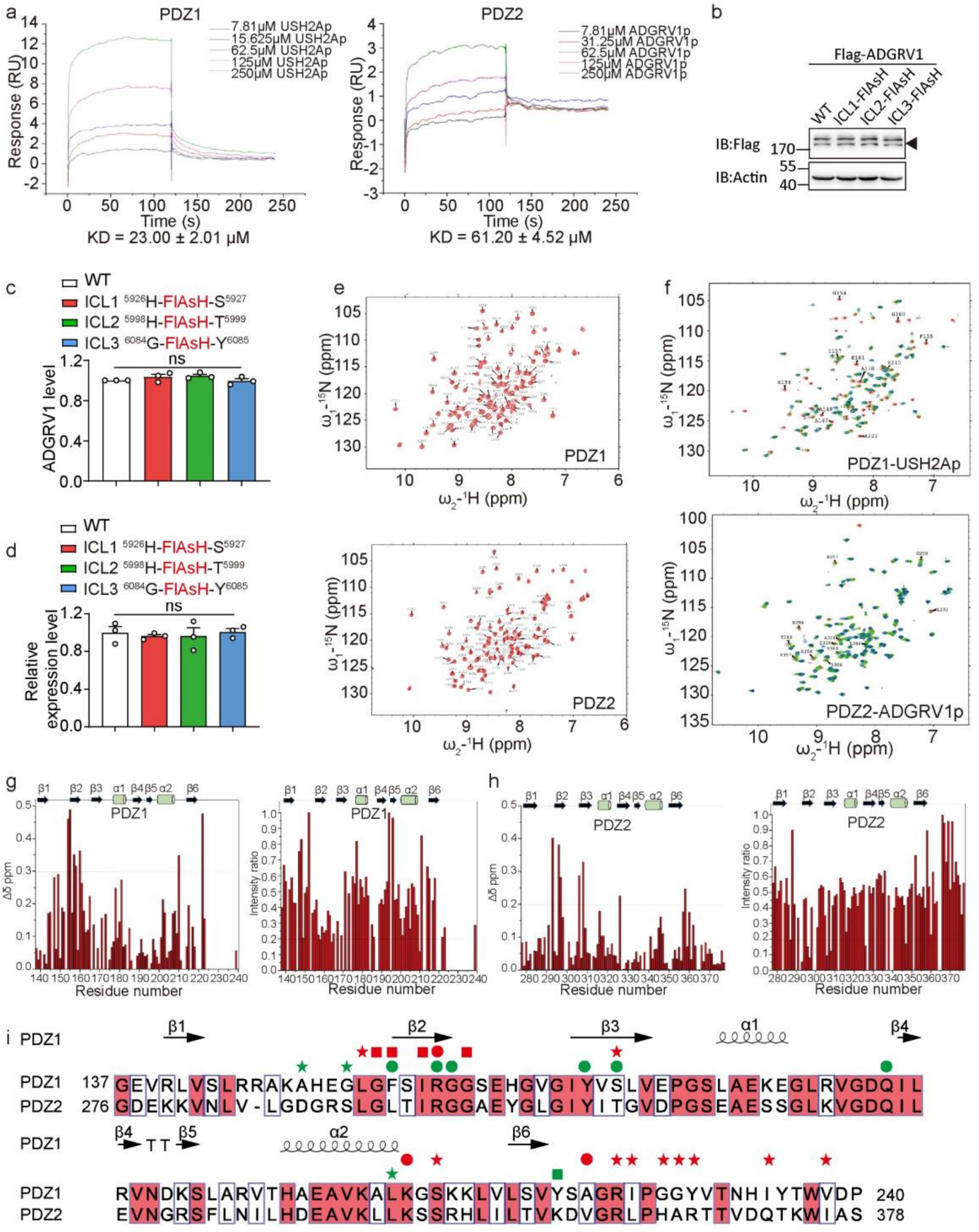

Figure s7. Delineating the interaction mode between ADGRV1/USH2A and WHRN by using SPR and NMR analysis.

a) Surface plasmon resonance (SPR) analysis of the binding of an increasing amount of USH2Ap or ADGRV1p to the PDZ1 or PDZ2 of WHRN. The increase in RUs from baseline was measured and used to calculate the affinity constant (KD).

b) Representative blotting showing the expression levels of WT ADGRV1 and ADGRV1-FlAsH mutants in HEK293 cells transfected with equal amounts of plasmids encoding Flag-

tagged ADGRV1 or its FlAsH mutants.

c) Relative ADGRV1 and its FlAsH mutants levels in HEK293 cells. Data are correlated to Fig. s7b and are normalized to the WT ADGRV1 level (normalized to 1). Data are from three independent experiments (n=3).

d) Relative cell-surface expression levels of WT ADGRV1 and ADGRV1-FlAsH mutants in HEK293 cells transfected with equal amounts of plasmids encoding Flag-tagged ADGRV1 or FlAsH mutants. Data are obtained from cell-surface ELISA assay and are normalized to the WT ADGRV1 level (normalized to 1). Data are from three independent experiments (n=3).

e) 600 MHz ^{1}H-^{15}N HSQC spectra of WHRN-PDZ1 and WHRN-PDZ2 domain.

f) Upper panel: Chemical shift perturbation of WHRN-PDZ1 domain titrated with USH2Ap. The WHRN-PDZ1 without peptide is shown in red and the last point (the molar ratio of PDZ1/Ush2Ap=1:4) of the titration in blue. The intermediate points are shown in shades of green. Lower panel: Chemical shift perturbation of WHRN-PDZ2 domain titrated with ADGRV1p. The WHRN-PDZ2 without peptide is shown in red and the last point (the molar ratio of PDZ2/ADGRV1p=1:5) of the titration in blue. The intermediate points are shown in shades of green.

g) The chemical shift change (Left panel) and resonance intensity reduction (Right panel) of WHRN-PDZ1 between the first and last titration point when titrated with USH2Ap. The horizontal dashed lines represent the significance level of chemical shift change > 0.3 ppm and intensity ratio < 20%, respectively.

h) The chemical shift change (Left panel) and resonance intensity reduction (Right panel) of WHRN-PDZ2 between the first and last titration point when titrated with ADGRV1p. The horizontal dashed lines represent the significance level of chemical shift change > 0.2 ppm and intensity ratio < 20%, respectively.

i) Sequence alignments of WHRN-PDZ1 domain with WHRN-PDZ2 domain. Circles represent the residues with significant changes both in chemical shift and resonance intensity. Squares indicate the residues with chemical shift change only. Five-pointed stars represent the residues with resonance intensity reduction only.

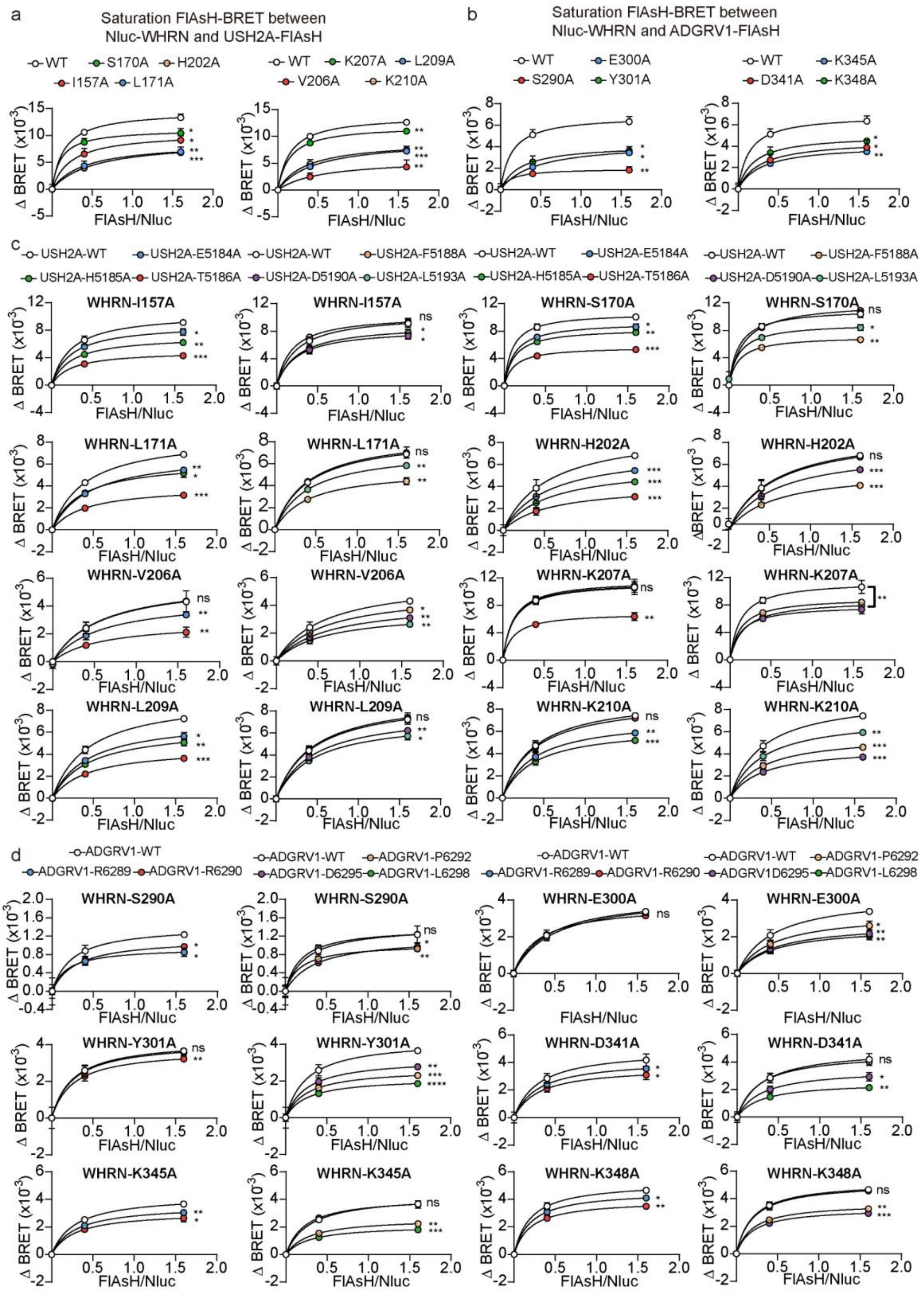

Figure s8. Identifying the potential hot spot interactions between ADGRV1/USH2A and WHRN by FlAsH-BRET assay.

a) Effect of WHRN-PDZ1 mutations at the putative binding sites on the saturation BRET signal between Nluc-tagged WHRN and USH2A-FlAsH. All the WHRN mutants showed decreased

saturation BRET signal compared with the WT WHRN when interacting with the USH2A-FlAsH. Based on these results, the pairing of WHRN mutants with USH2A (WT and mutants) through alanine scanning and FlAsH-BRET assay were subsequently performed to identify the potential hot spot interactions between WHRN and USH2A (Fig.s8c).

b) Effect of WHRN-PDZ2 mutations at the putative binding sites on the saturation BRET signal between Nluc-tagged WHRN and ADGRV1-ICL3-FlAsH. All the WHRN mutants showed decreased saturation BRET signal compared with the WT WHRN when interacting with the ADGRV1-FlAsH. Based on these results, the pairing of WHRN mutants with ADGRV1 (WT and mutants) through alanine scanning and FlAsH-BRET assay were subsequently performed to identify the potential hot spot interactions between WHRN and ADGRV1 (Fig.s8d).

c) Effect of USH2A mutations at the putative binding sites with WHRN-PDZ1 on the saturation BRET signal between Nluc-tagged WHRN and USH2A-FlAsH. For a given WHRN mutant (I157A, S170A, L171A, H202A, V206A, K207A, L209A or K210A), the USH2A mutants that did not show significantly decreased BRET signal compared to the WT USH2A when interacting with this specific WHRN mutant are deduced to be potential hot spot interaction pairs. Representative curves from three independent experiments were shown.

d) Effect of ADGRV1 mutations at the putative binding sites with WHRN-PDZ2 on the saturation BRET signal between Nluc-tagged WHRN and USH2A-FlAsH. For a given WHRN mutant (S290A, E300A, Y301A, D341A, K345A or K348A), the ADGRV1 mutants that did not show significantly decreased BRET signal compared to the WT ADGRV1 when interacting with this specific WHRN mutant are deduced to be potential hot spot interaction pairs. Representative curves from three independent experiments were shown.

a, b) *$P < 0.05$; **$P < 0.01$; ***$P < 0.001$; WHRN mutants compared with WT WHRN when interacting with the USH2A-FlAsH. c) *$P < 0.05$; **$P < 0.01$; ***$P < 0.001$; ns, no significant difference. USH2A mutants compared with WT USH2A when interacting with a given WHRN mutant. d) *$P < 0.05$; **$P < 0.01$; ***$P < 0.001$; ns, no significant difference. ADGRV1 mutants compared with WT ADGRV1 when interacting with a given WHRN mutant. The bars indicate the mean ± SEM values. The saturating BRET data were statistically analyzed using one-way ANOVA with Dunnett’s post hoc test.
